# Supplementary material for: Circularly polarized OLEDs from chiral plasmonic nanoparticle-molecule hybrids
Source: Nat Commun. 2025 Feb 15;16:1658. doi: 10.1038/s41467-025-57000-8 (PMC11830063; doi:10.1038/s41467-025-57000-8)
Supplement: Supplementary file 1 — Supplementary Information [file 41467_2025_57000_MOESM1_ESM.pdf]

Supplementary information

Circularly polarized OLEDs from chiral plasmonic nanoparticle-molecule hybrids

Jiapeng Zheng[1,2], Yuang Fu[2], Jing Wang[3], Wei Zhang[4], Xinhui Lu[2], Hai-Qing Lin[5], Lei Shao[3]* & Jianfang Wang[2]*

[1]School of Artificial Intelligence Science and Technology, Institute of Photonic Chips, University of Shanghai for Science and Technology, Shanghai 200093, China.
[2]Department of Physics, The Chinese University of Hong Kong, Shatin, Hong Kong SAR 999077, China.
[3]State Key Laboratory of Optoelectronic Materials and Technologies, Guangdong Provincial Key Laboratory of Display Materials and Technologies, School of Electronics and Information Technology, Sun Yat-sen University, Guangzhou, Guangdong 510275, China.
[4]National Key Laboratory of Computational Physics, Institute of Applied Physics and Computational Mathematics, Beijing 100088, China.
[5]School of Physics, Zhejiang University, Hangzhou, Zhejiang 310058, China.

*Corresponding author. E-mail: shaolei5@mail.sysu.edu.cn; jfwang@phy.cuhk.edu.hk

This PDF file includes:

Supplementary Figures 1 to 34

Supplementary Table 1

Supplementary References 1 to 82

Supplementary Table 1 | Comparison of different CP-OLED devices

Emissive layer	g_{EL}	$\|g_{EL}\|$	EQE_{max} (%)	Supplementary reference
CP-OLED-1	≈ 0.15	≈ 0.15	1.3	This work
CP-OLED-2	0.31	0.31	2.5	This work
CP-OLED-3	0.32	0.32	2.1	This work
30 wt% *S*-BACzBO:PPF	-5.3×10^{-4}	0.00053	36.8	1
M,*M*-RBNN	$+1.91\times10^{-3}$	0.00191	36.6	2
P-BN[9]H	-6.2×10^{-3}	0.0062	35.4	3
R-CzOBN:POT2T:BN1	$+2.8\times10^{-3}$	0.0028	33.2	4
(*R*)-Czp-tBuCzB	$+1.54\times10^{-3}$	0.00154	32.1	5
TCTA:*R*-TRZOBN:Ir1	$+3.2\times10^{-3}$	0.0032	32	6
Ir*R*	$+2.6\times10^{-3}$	0.0026	31.9	7
(*S*)-OBN-Cz	2.30×10^{-3}	0.0023	31.7	8
(M)-helicene-BN	-2.2×10^{-3}	0.0022	30.7	9
R-NPACZ:(tfmppy)$_2$Ir(pop)	$+2.2\times10^{-3}$	0.0022	30.7	10
(*P*)-BN-Py	-4.37×10^{-4}	0.000437	30.6	11
Λ-Ir-(*R*-camphor)	-4.36×10^{-4}	0.000436	30.5	12
(+)-(*S*)-ax-DMAC	-2.0×10^{-3}	0.002	30.1	13
(*R*)-OBN-2CN-BN	$+1.43\times10^{-3}$	0.00143	29.4	14
3 wt% Ir(tptpy)2acac:(*S*)-BNPCN-p-CP	-2.32×10^{-3}	0.00232	29.3	15
(*R*)-ODQPXZ	-6.0×10^{-4}	0.0006	28.3	16
D-(R)-BPSPXZ	-8.5×10^{-3}	0.0085	28.5	17
(*M*)-DB-O	$+2.2\times10^{-3}$	0.0022	27.5	18
R-DOBNT	-1×10^{-3}	0.001	25.6	19
(*S*)-SCFPY	$+3.6\times10^{-3}$	0.0036	23.3	20
(*S*)-SFOT	$+1.0\times10^{-3}$	0.001	23.1	21
(*R*,*R*)-pTpAcBP	-1.0×10^{-3}	0.001	22.1	22

R/S-(BINAP)$_2$Cu(μ-I$_2$)	3.0×10^{-3}	0.003	21.7	23
(*S*,*S*)-CPAD	−1.4×10^{-3}	0.0014	21.5	24
R-Ax-CN	+4.2×10^{-3}	0.0042	21	25
(*S*,*S*)-*TpAc-TRZ* (undoped devices)	+1.5×10^{-3}	0.0015	20.7	26
(+)-BN4	+3.7×10^{-3}	0.0037	20.6	27
(*R*)-SPOCN+(*S*)-OSFSO	+2.5×10^{-3}	0.0025	20.4	28
(*R*)-TRZ-MeIAc	+6.4×10^{-4}	0.00064	20.3	29
(*M*,*M*)-CNSPZ	+2.9×10^{-3}	0.0029	20.03	30
(*S*)-OSFSO	+3.1×10^{-3}	0.0031	20	31
(+)-(*S*,*S*)-CAI-Cz	−1.7×10^{-3}	0.0017	19.7	32
(*R*)-OBN-3CN	−1.1×10^{-3}	0.0011	19.7	33
R-OBN-AICz	+4.7×10^{-4}	0.00047	19	34
S-PXZ-PT	−1.3×10^{-3}	0.0013	18.5	35
(*R*)-P-BPCZ4	−5.5×10^{-3}	0.0055	18.3	36
(*S*)-BPPOACZ	+4.5×10^{-3}	0.0045	17.8	37
(+)-(*R*,*R*)-MC	+1.5×10^{-3}	0.0015	17.1	38
(*R*)-BN-2Mcp: Ir(mppy)$_3$	−1.3×10^{-3}	0.0013	17.1	39
S-OBS-Cz	−1×10^{-3}	0.001	17	40
P-Pt	−1.6×10^{-3}	0.0016	16.26	41
S-P	+1.6×10^{-3}	0.0016	15.8	42
λ-Ir(dfppy)$_2$(*S*-sdpp)	−2.1×10^{-3}	0.0021	14.6	43
(*P*)-QAO-PhCz	+1.5×10^{-3}	0.0015	14	44
R_p-MAC*-Cu-CzP	+4.5×10^{-4}	0.00045	13.2	45
TAPC:*R*-TRZ	+7.25×10^{-3}	0.00725	12.7	46
(−)-(*S*)-Cz-Ax-CN	−1.2×10^{-2}	0.012	12.5	47
(*S*)-OBN-tBuCz	+1.57×10^{-3}	0.00157	12.4	48

SO-D3	$+6\times10^{-4}$	0.0006	12.4	49
R-OBN-DPA	$+2.3\times10^{-3}$	0.0023	12.3	50
R-pSACODP	-1.1×10^{-3}	0.0011	12	51
D-(*R*)-C-DpCpN-Trz	$+7.6\times10^{-4}$	0.00076	11.3	52
R-Pt	0.06	0.06	11.3	53
(*M*)-QPO-PhCz	$+1.6\times10^{-3}$	0.0016	10.6	54
(*R*)-BIPNX-TRZ	-8.4×10^{-4}	0.00084	10.2	55
S-CPDCz	-3.7×10^{-3}	0.0037	10.1	56
R-$Ag_6(PTLT)_6$	-5.3×10^{-3}	0.0053	10	57
S-BN-CF	0.026	0.026	9.3	58
S_p-5	$+4.3\times10^{-3}$	0.0043	7.8	59
(*R*)-CO-PhDPA	-2.4×10^{-3}	0.0024	6.4	60
R-P	$+1.6\times10^{-3}$	0.0016	6.2	61
D-(*S*,*S*)-DCz	-8.3×10^{-4}	0.00083	5.5	62
$(S\text{-}2Cz)_{0.2}$-$(PFpy)_{0.8}$-$(Ir(MDQ)_2)_{0.1}$	−0.014	0.014	4.1	63
(Λ)-Ir2((−)-CS)/(−)-IL	-2.5×10^{-3}	0.0025	4.09	64
R-5 (undoped devices)	$+5.6\times10^{-3}$	0.0056	2.79	65
L-HP-NTi	−0.023	0.023	2.3	66
(*R*)-BP2	$+1.11\times10^{-3}$	0.00111	2.15	67
$[Sm(tta)_3$(d-phen)]	0.011	0.011	1.55	68
(*R*)/(*S*)-[tmd]	3.0×10^{-3}	0.003	1.4	69
R-3	-9.8×10^{-3}	0.0098	1.2	70
R-P37+f8bt	−0.02	0.02	1.08	71
S-BN-tCz	2.13×10^{-3}	0.00213	1	72
(*R*)-C′3	1.0×10^{-3}	0.001	0.8	73
$(S\text{-}M)_{0.2}$-$(BP)_{0.8}$	0.01	0.01	0.69	74

F8BT+*S*-6	-1.86×10^{-2}	0.0186	0.54	75
$(S\text{-P2})_{0.6}$-$(\text{NPy})_{0.4}$	$+4.6\times10^{-2}$	0.046	0.21	76
M-H6$(\text{TMS})_2$	8.0×10^{-3}	0.008	0.21	77
CsEu((-)–hfbc)$_4$	−1	1	0.05	78
Ln(III) complexes	0.51	0.51	0.48	79
CsEu((+)–hfbc)$_4$	0.73	0.73	0.0042	80
CsEu((-)–hfbc)$_4$	−0.61	0.61	0.002	78

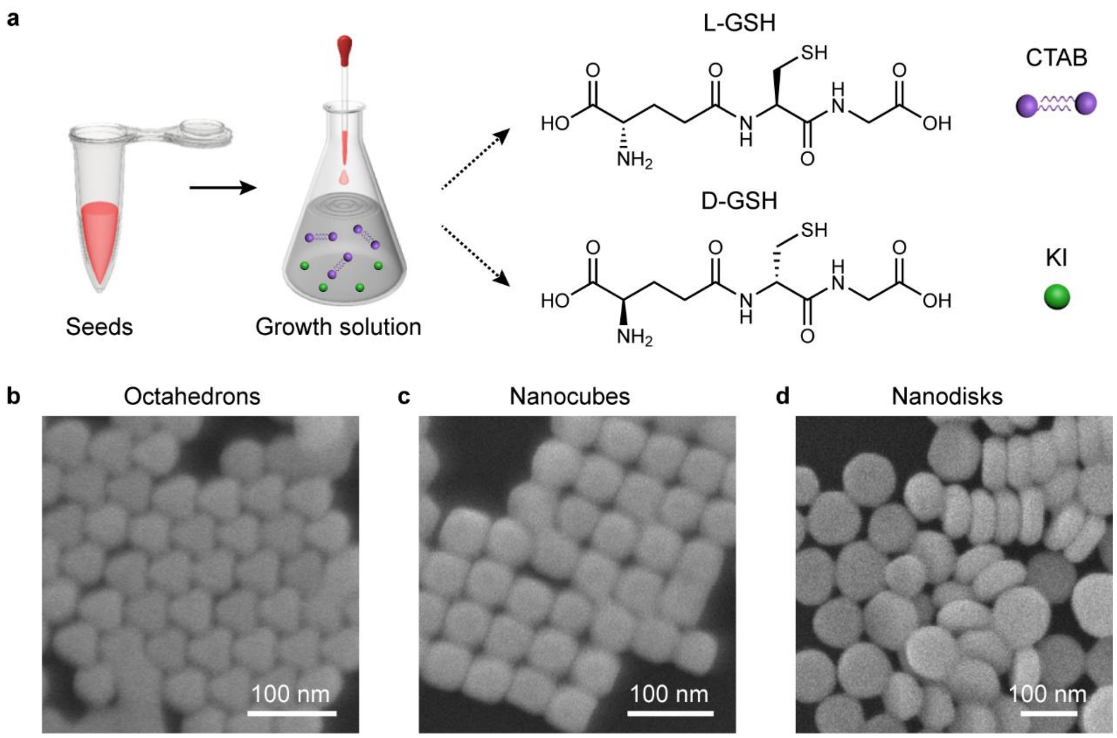

Supplementary Fig. 1 | Synthesis of chiral plasmonic NPs. a Schematic showing the process of growing chiral plasmonic NPs from seeds. The prepared Au nanocrystal seeds were added into the growth solution consisting of GSH, CTAB, KI, $HAuCl_4$, and AA. **b**–**d** Scanning electron microscopy (SEM) images of the different Au seeds, including octahedrons with side lengths of 33 nm (**b**), nanocubes with side lengths of 45 nm (**c**), and nanodisks with diameters of 100 nm and thicknesses of 30 nm (**d**). The GSH-directed and halide-assisted differential growth strategy was employed to guide the chiral growth of the Au octahedrons, nanocubes, and nanodisks into the 432 helicoid III, 432 helicoid IV, and nanotriskelions, respectively.

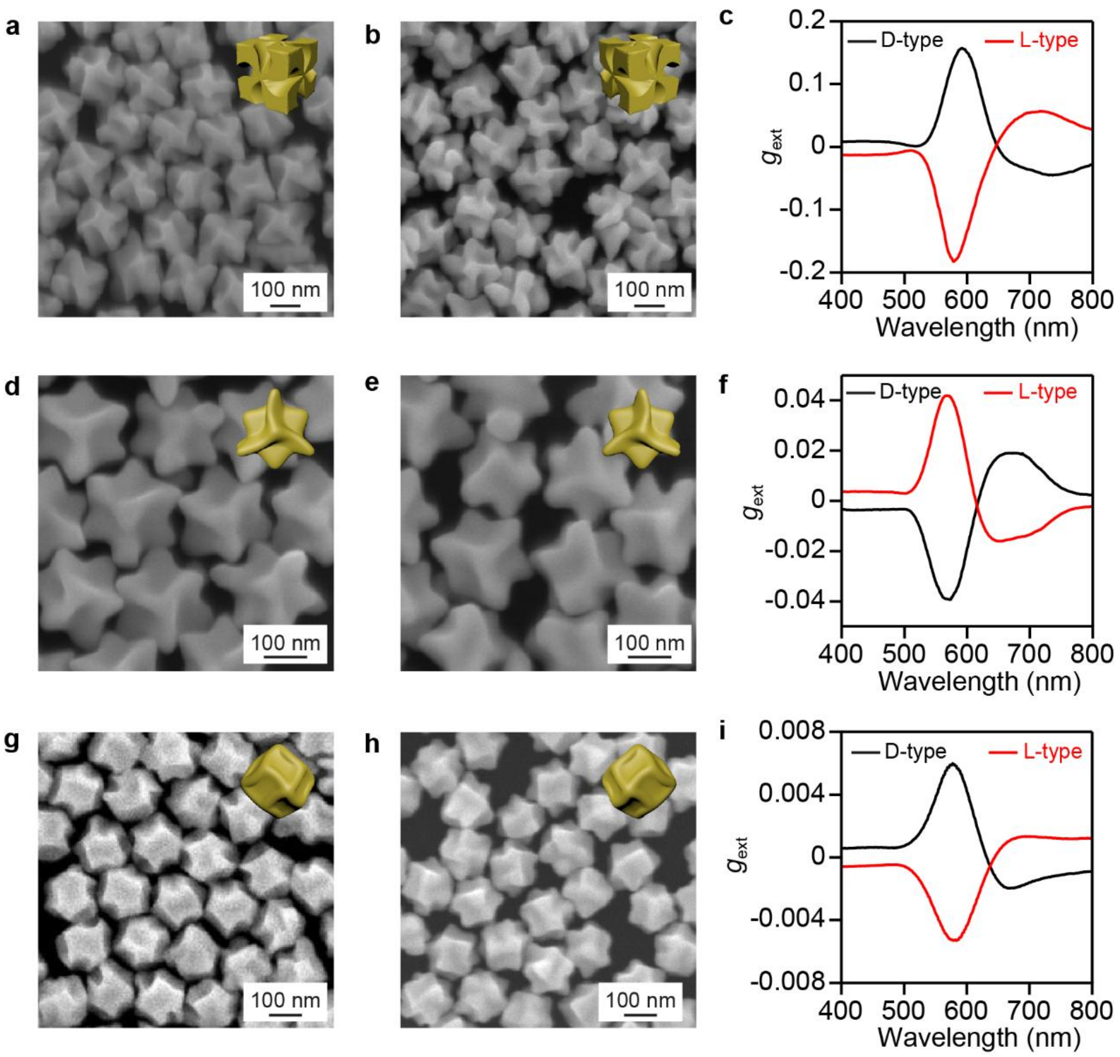

Supplementary Fig. 2 | Morphologies and extinction dissymmetry factor spectra of the chiral plasmonic NPs. a–c SEM images (**a**,**b**) and extinction dissymmetry factor (g_{ext}) spectra (**c**) of the 432 helicoid III NPs grown in the presence of L- and D-GSH. **d–f** SEM images (**d**,**e**) and g_{ext} spectra (**f**) of the nanotriskelions grown in the presence of L- and D-GSH. **g–i** SEM images (**g**,**h**) and g_{ext} spectra (**i**) of the 432 helicoid IV NPs grown in the presence of L- and D-GSH. The opposite geometric chirality between the L-432 helicoid III NPs along the <100> directions and the L-nanotriskelions along the <111> directions result from the halide-assisted favored growth along different crystal axes in the presence of the same L-type chiral ligand. Such varying geometric chirality gives rise to the inverted chiroptical responses from the two types of chiral NPs. The 432 helicoid IV NPs show a very weak chiroptical response because of their opposite structural chirality along the <111> and <100> directions. The enantiomeric NPs were grown from L and D-GSH, exhibiting mirror-symmetric nanostructures and opposite chiroptical activities.

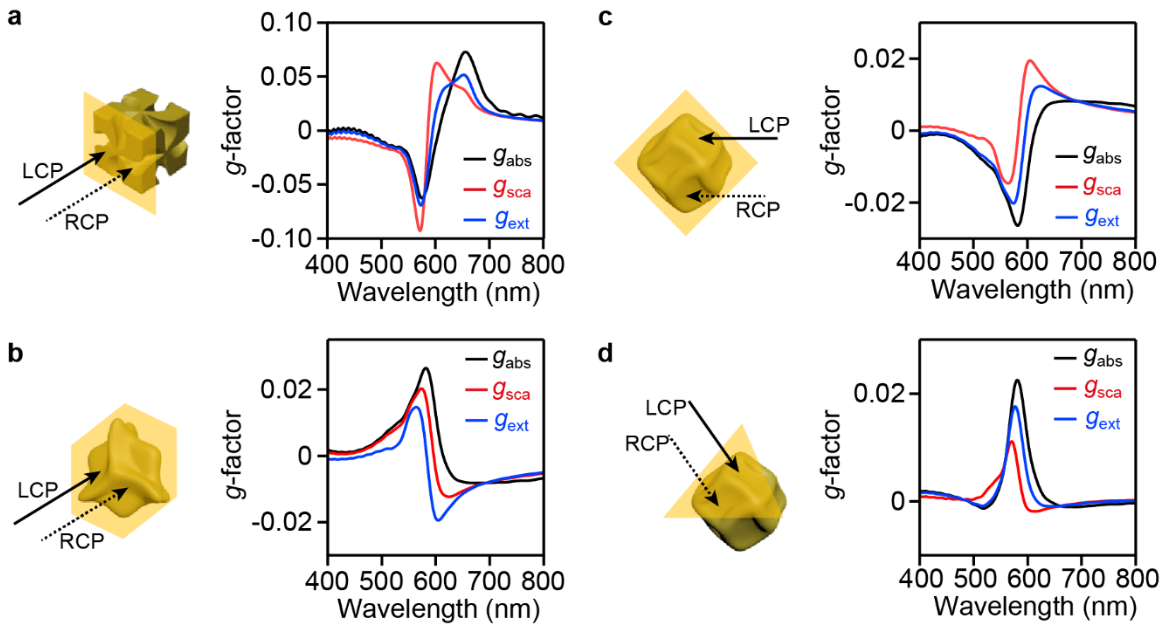

Supplementary Fig. 3 | FDTD-simulated chiroptical responses of the chiral plasmonic NPs. a Simulated g_{abs}, g_{sca}, and g_{ext} spectra of the L-432 helicoid III NP under the excitation of light incident along the <100> directions. **b** Simulated g_{abs}, g_{sca}, and g_{ext} spectra of the L-nanotriskelion under the excitation of light incident along the <111> directions. **c**,**d** Simulated g_{abs}, g_{sca}, and g_{ext} spectra of the L-432 helicoid IV NP under the excitation of light incident along the <100> (**c**) and <111> (**d**) directions. The simulations demonstrate the opposite chiroptical activities between the L-432 helicoid III NP and L-nanotriskelion, as well as the weak chiroptical activity of the L-432 helicoid IV NP.

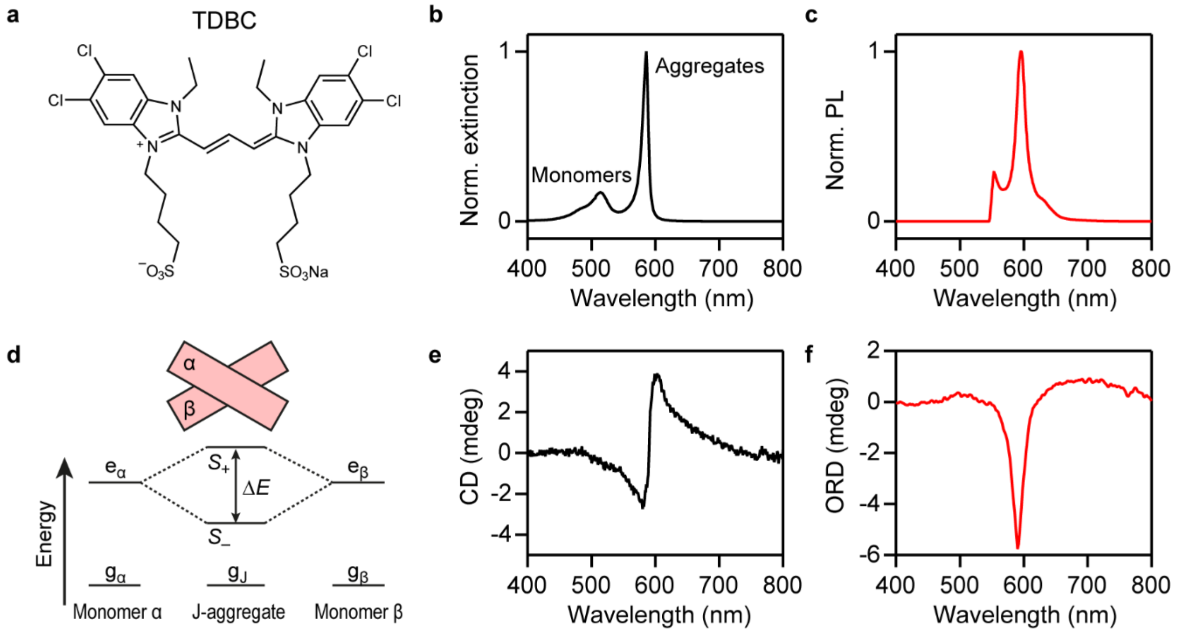

Supplementary Fig. 4 | Monomer molecular structure and optical properties of the TDBC J-aggregate film. a–c Molecular structure of TDBC monomer (**a**), extinction (**b**), and photoluminescence (PL) spectra (**c**) of the TDBC J-aggregate film. The TDBC J-aggregate film exhibits a narrow absorption band at 585 nm and an emission peak at 600 nm. **d** Energy level diagrams showing the generation of chiral TDBC excitons from interchromophoric interaction. **e**,**f** CD spectra (**e**) and optical rotatory dispersion (ORD) spectra (**f**) of the TDBC J-aggregate film. The TDBC J-aggregate film exhibits a bisignate CD signal and ORD dip at the absorption energy.

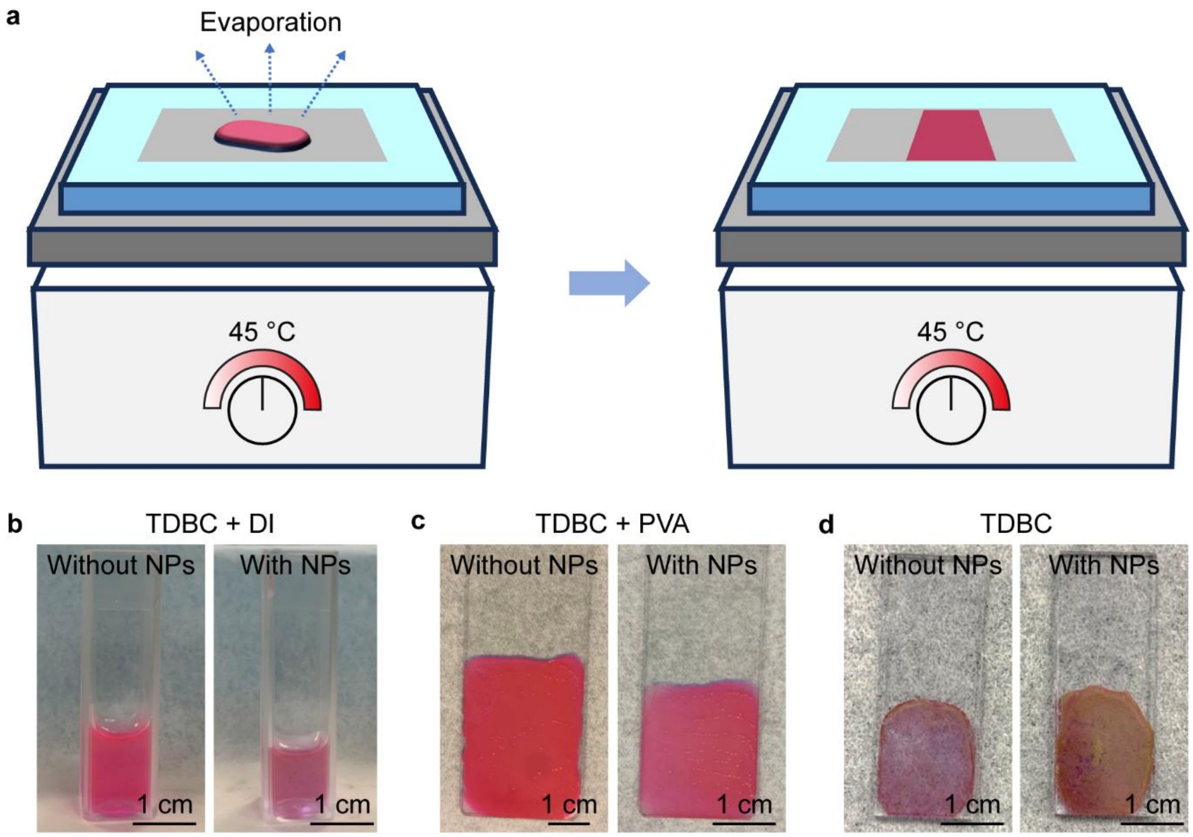

Supplementary Fig. 5 | Fabrication of (plasmonic NP)–(TDBC aggregate) hybrid films. **a** Schematic diagram showing the evaporation-driven assembly of TDBC aggregates on the plasmonic NPs. **b** Photographs of TDBC in solution without and with the chiral NPs. **c** Photographs of TDBC in PVA matrices without and with the chiral NPs. **d** Photographs of the TDBC aggregate films without and with the chiral NPs.

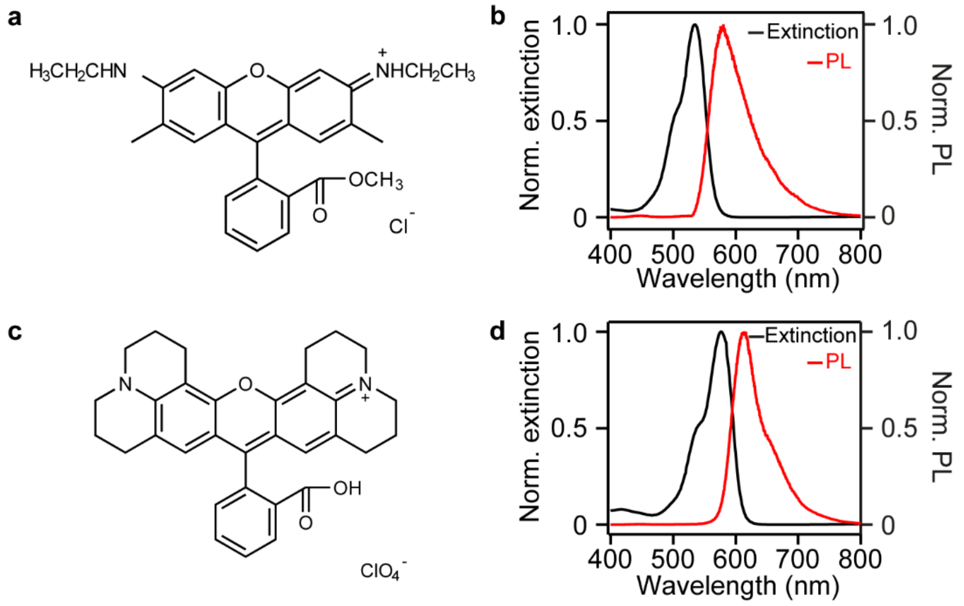

Supplementary Fig. 6 | Molecular structures and optical properties of different chromophores. a,b Molecular structure of R590 (**a**), extinction (black line, **b**) and PL spectra (red line, **b**) of the R590 film. R590 exhibits an absorption band at 525 nm and an emission peak at 580 nm. **c,d** Molecular structure of R640 (**c**), extinction (black line, **d**) and PL spectra (red line, **d**) of the R640 film. R640 exhibits an absorption band at 575 nm and an emission peak at 615 nm.

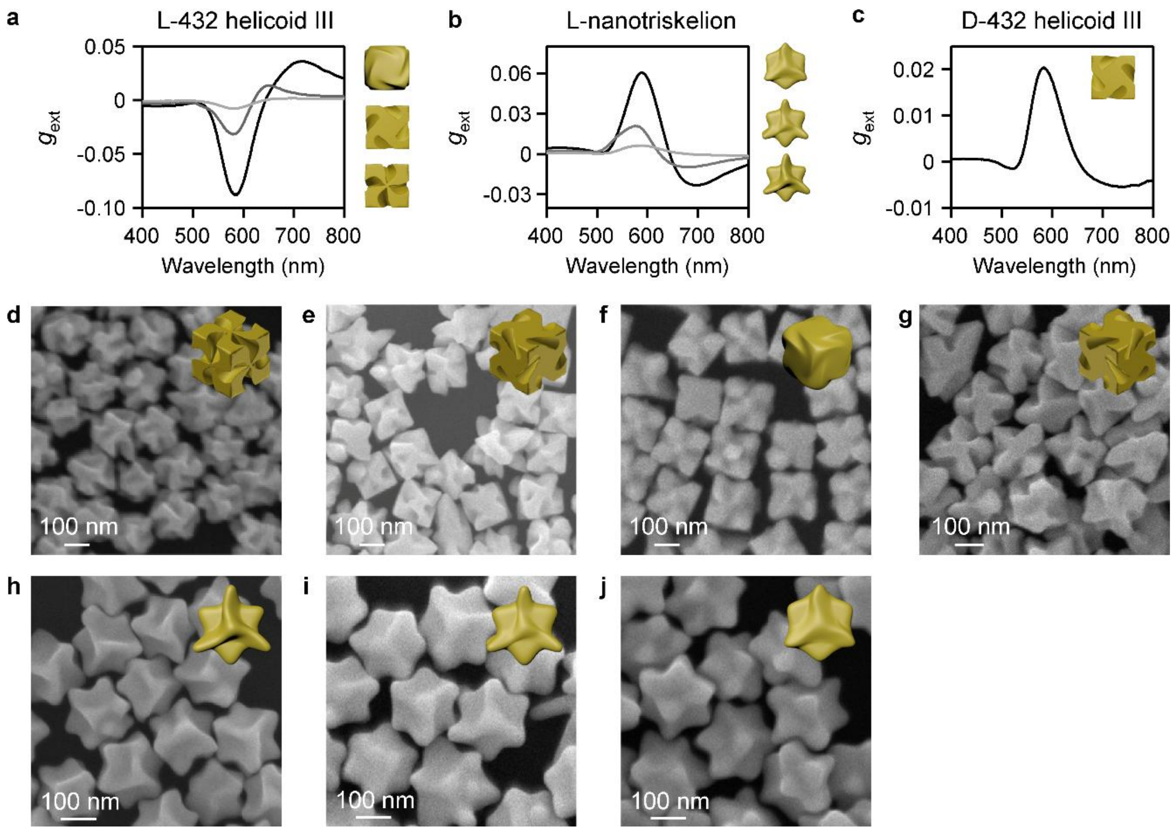

Supplementary Fig. 7 | Libraries of the chiral plasmonic NPs with varying geometric chiral dissymmetries in Fig. 2. a–**c** g_{ext} spectra of the chiral plasmonic NPs. **d**–**f** SEM images of the L-432 helicoid III NPs, with their g_{ext} spectra shown in (**a**). **g** SEM image of the D-432 helicoid III NPs, with their g_{ext} spectrum shown in (**c**). **h**–**j** SEM images of the nanotriskelions, with their g_{ext} spectra shown in (**b**). These chiral plasmonic NPs were synthetically designed to have varying geometric chiral dissymmetries.

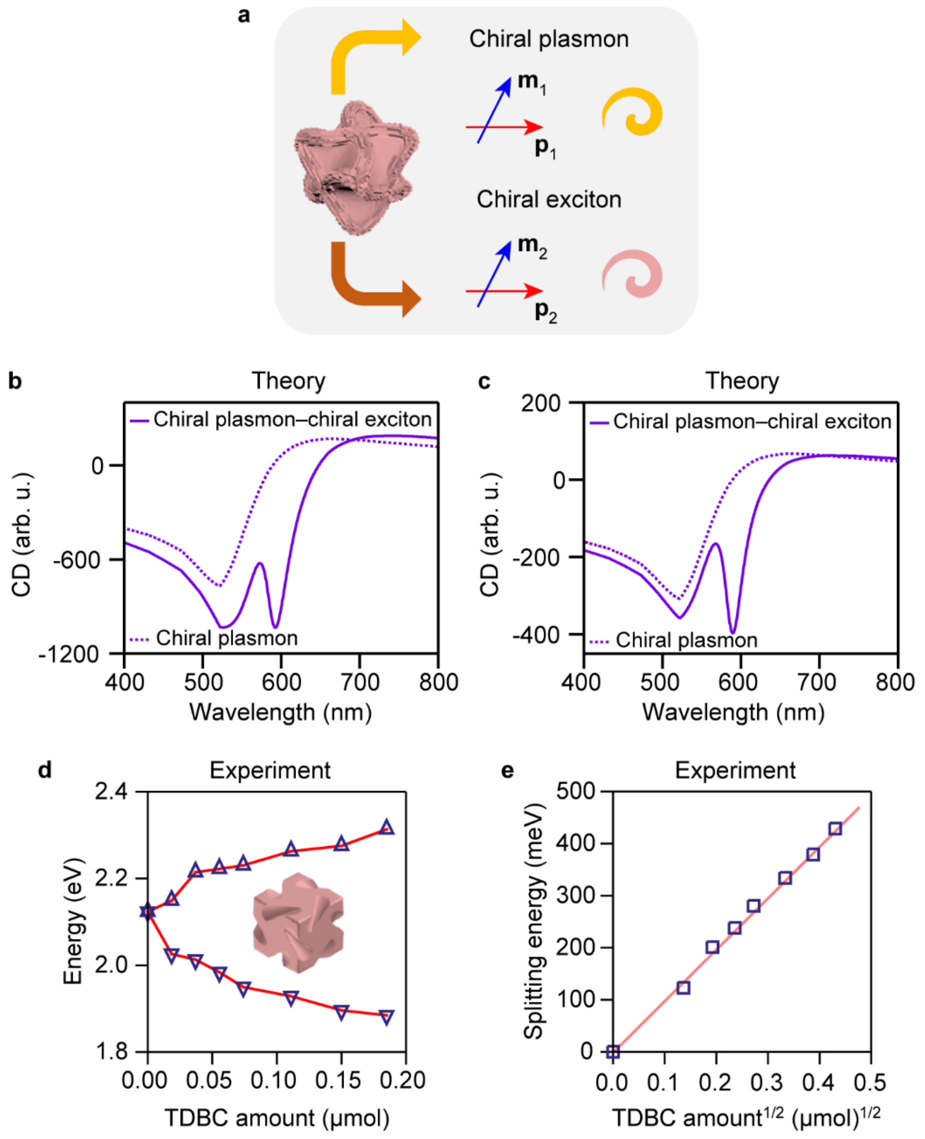

Supplementary Fig. 8 | Calculated chiroptical responses of the coupled chiral plasmonic–excitonic systems. a Schematic showing the chiral-plasmon–chiral-exciton system. The TDBC aggregates were assembled on the surface of the chiral NPs in a chiral configuration, endowing the hybrid structure with both chiral plasmons and chiral excitons. Both chiral plasmons and excitons can be described by effective electric and magnetic dipoles. Their interaction finally results in various chiroptical responses. **b**,**c** Chiroptical responses of the hybrid NP–molecule structures calculated from our theoretical model. NPs with different chiroptical responses were modeled. The CD spectra show distinct peak/dip splitting. The theoretical model also indicates the CD inversion around the dip at 590 nm, consistent with the experimental observation. **d**,**e** Energy splitting of the extinction CD spectra of the (D-432 helicoid III NP)–(TDBC aggregate) hybrids shown in Fig. 2g in the main text. The energy splitting is gradually enlarged from 20 meV to 430 meV (**d**) as the initial TDBC amount is increased from 18.5 nmol to 185 nmol, showing the evolution of the (chiral plasmon)–(chiral exciton) interaction from weak- to strong-coupling-like regimes. We further found that the energy splitting scales with the square root of the TDBC molecule concentration (**e**).

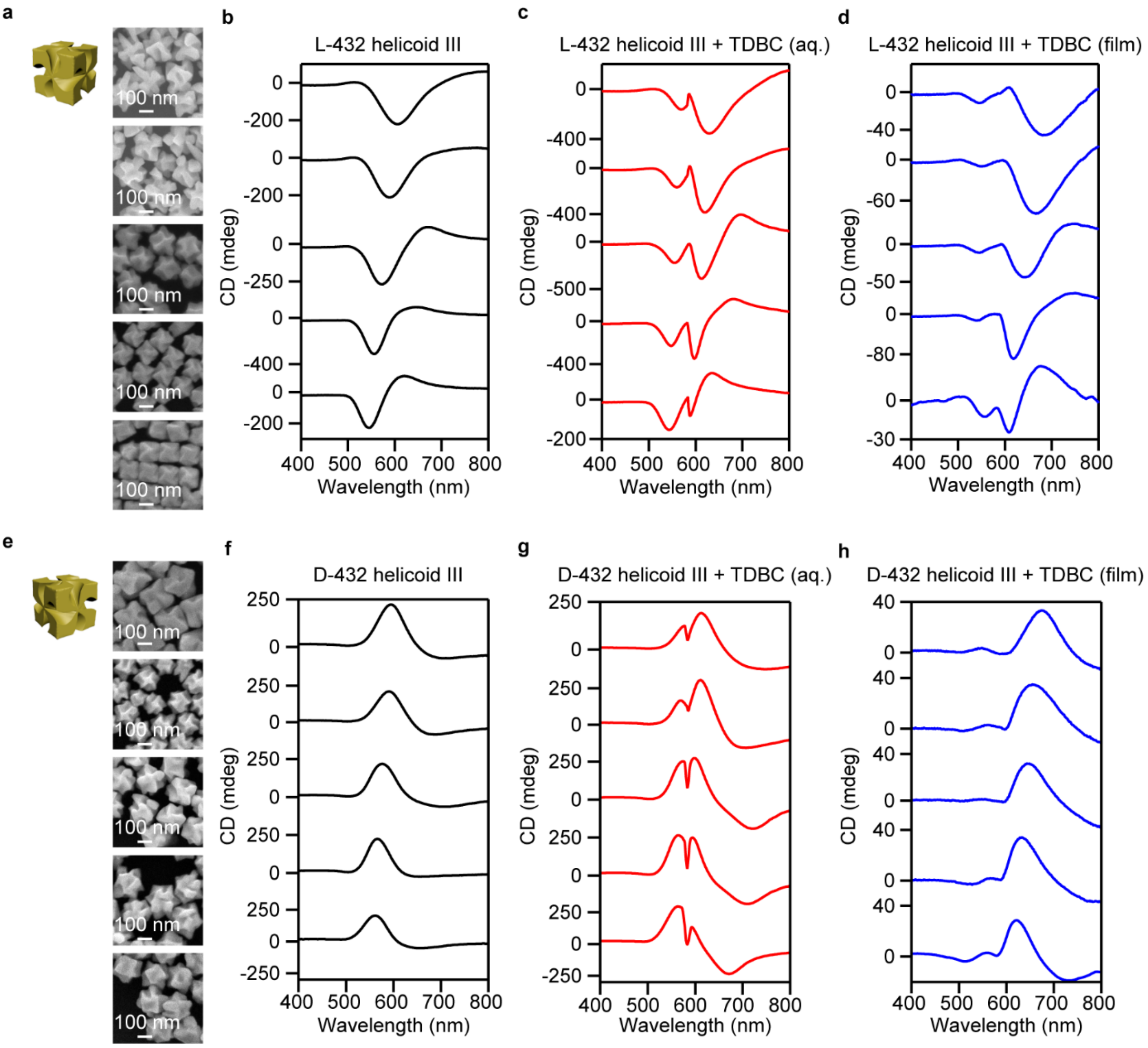

Supplementary Fig. 9 | Extinction CD spectra of the (432 helicoid III NP)–(TDBC aggregate) hybrids. a,**b** SEM images (**a**) and extinction CD spectra (**b**) of the L-432 helicoid III NPs. **c**,**d** Extinction CD spectra of the (L-432 helicoid III NP)–(TDBC aggregate) hybrids before (**c**) and after (**d**) the evaporation of water during the preparation of the hybrid films. **e**,**f** SEM images (**e**) and extinction CD spectra (**f**) of the D-432 helicoid III NPs. **g**,**h** Extinction CD spectra of the (D-432 helicoid III NP)–(TDBC aggregate) hybrids before (**g**) and after (**h**) the evaporation of water during the preparation of the hybrid films. The detuning between chiral plasmons and excitons can strongly affect the CD response of the (432 helicoid III NP)–(TDBC aggregate) hybrid solution, resulting in the splitting between an upper plexciton branch mode and a lower plexciton branch mode. After the evaporation of water, (432 helicoid III NP)–(TDBC aggregate) hybrid films were fabricated, causing more chiral excitons in the TDBC aggregates formed on the chiral NP surface. The chiral excitons have a chiroptical response around the extinction band of TDBC (585 nm). We then observed the enlarged splitting in the CD spectra when the hybrid system was changed from the aqueous solution to the solid film.

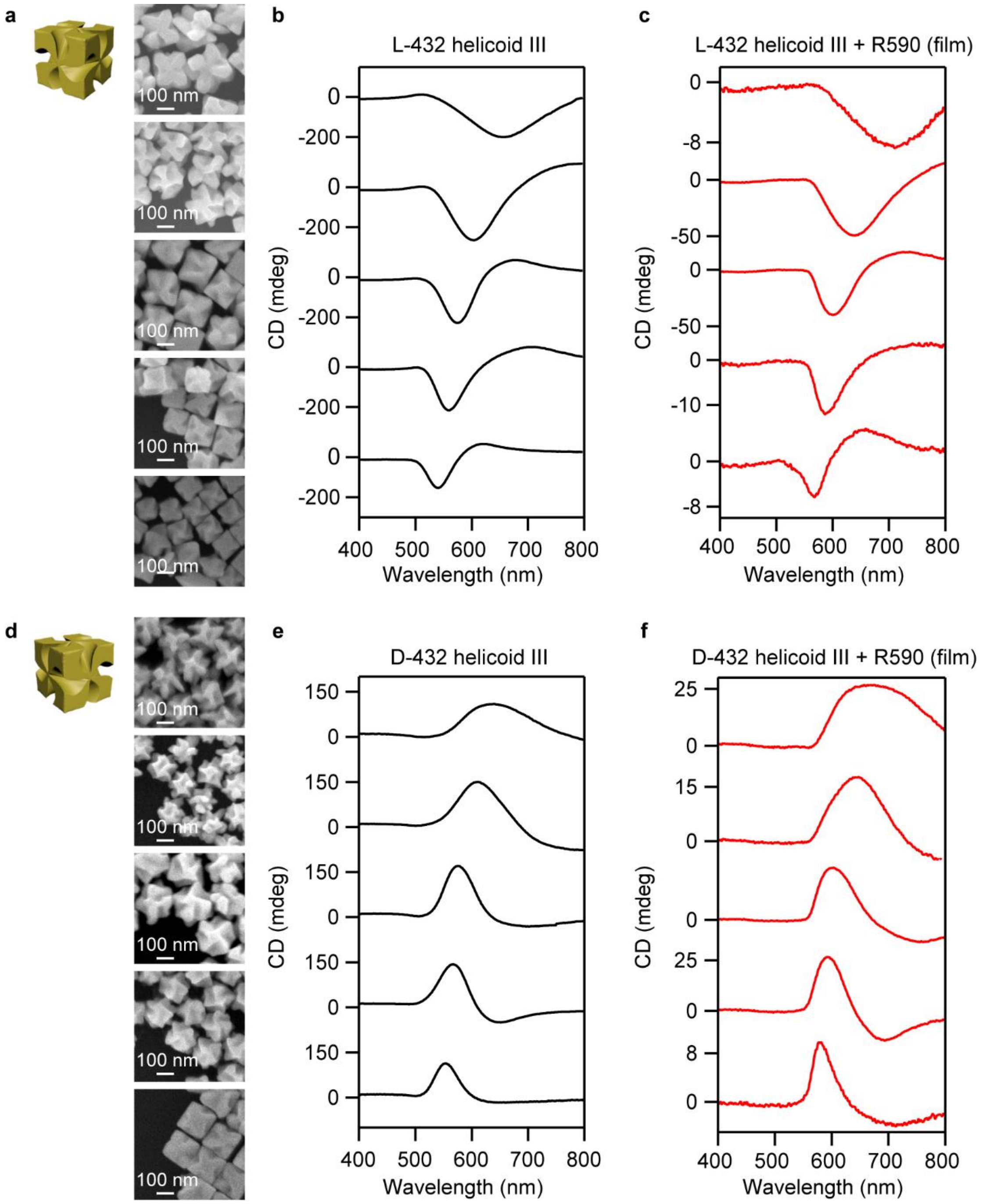

Supplementary Fig. 10 | Extinction CD spectra of the (432 helicoid III NP)–(R590) hybrids. a,**b** SEM images (**a**) and extinction CD spectra (**b**) of the L-432 helicoid III NPs. **c** Extinction CD spectra of the (L-432 helicoid III NP)–(R590) hybrid films prepared from the NPs shown in (**a**). **d**,**e** SEM images (**d**) and extinction CD spectra (**e**) of the D-432 helicoid III NPs. **f** Extinction CD spectra of the (D-432 helicoid III NP)–(R590) hybrid films prepared from the NPs shown in (**d**).

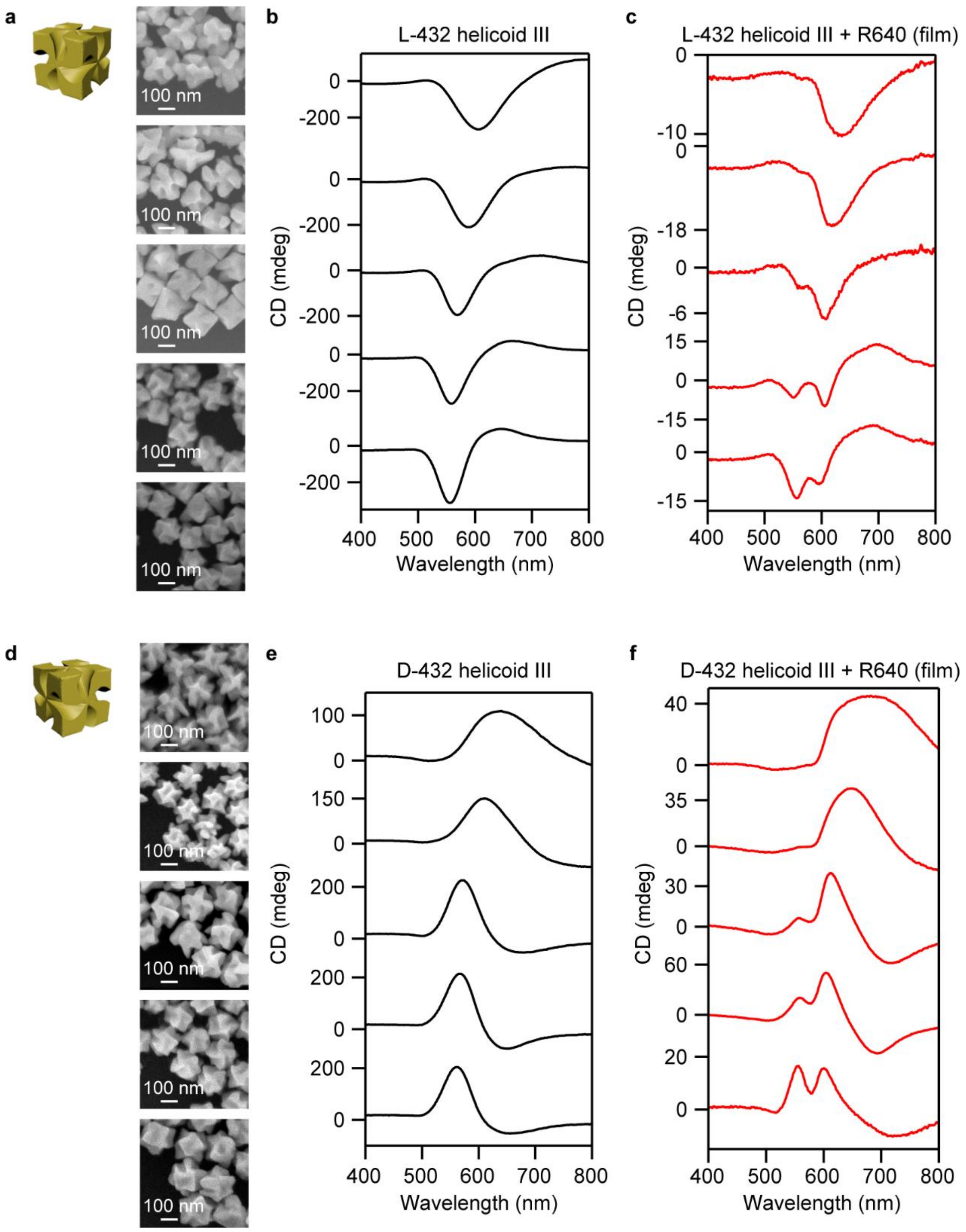

Supplementary Fig. 11 | Extinction CD spectra of the (432 helicoid III NP)–(R640) hybrids. a,**b** SEM images (**a**) and extinction CD spectra (**b**) of the L-432 helicoid III NPs. **c** Extinction CD spectra of the (L-432 helicoid III NP)–(R640) hybrid films prepared from the NPs shown in (**a**). **d**,**e** SEM images (**d**) and extinction CD spectra (**e**) of the D-432 helicoid III NPs. **f** Extinction CD spectra of the (D-432 helicoid III NP)–(R640) hybrid films prepared from the NPs shown in (**d**).

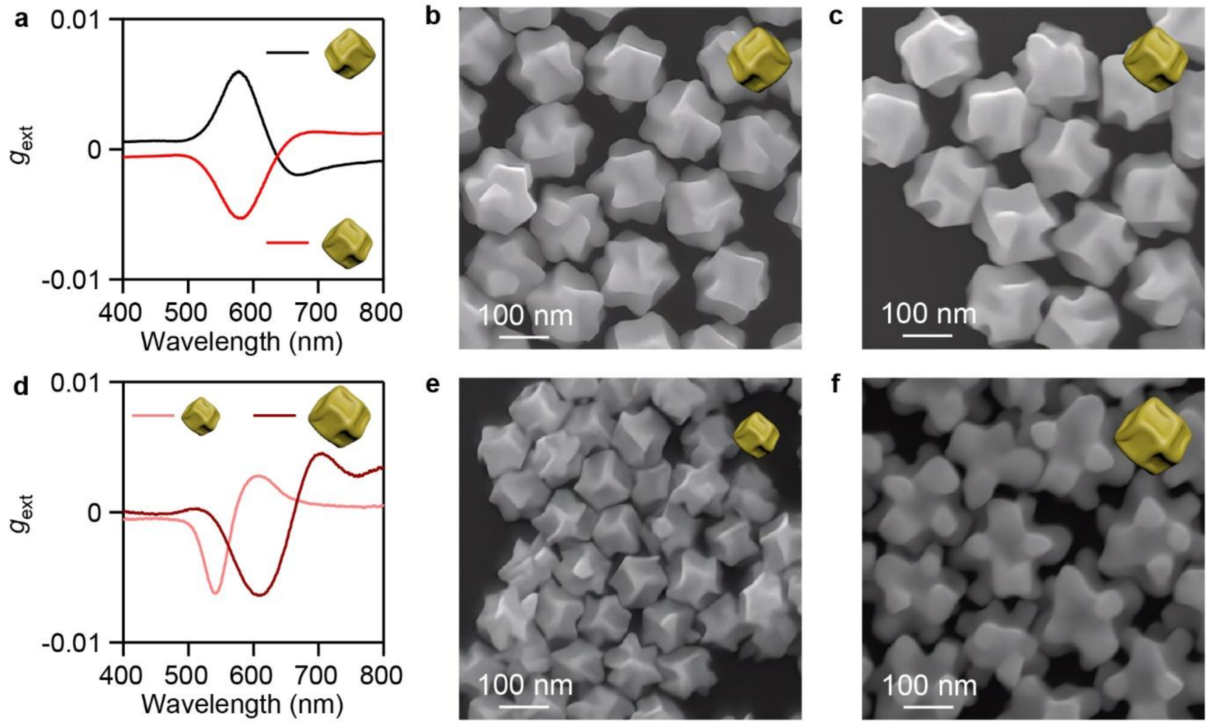

Supplementary Fig. 12 | 432 helicoid IV NPs with different handedness and dimensions in Fig. 3. a–c Extinction g_{ext} spectra of the 432 helicoid IV NPs (**a**), and SEM images of the D-type (**b**) and L-type (**c**) 432 helicoid IV NPs with the size D = 150 nm. **d–f** Extinction g_{ext} spectra of the 432 helicoid IV NPs (**d**), and SEM images of the L-432 helicoid IV NPs with the sizes D = 100 nm (**e**) and 210 nm (**f**), respectively.

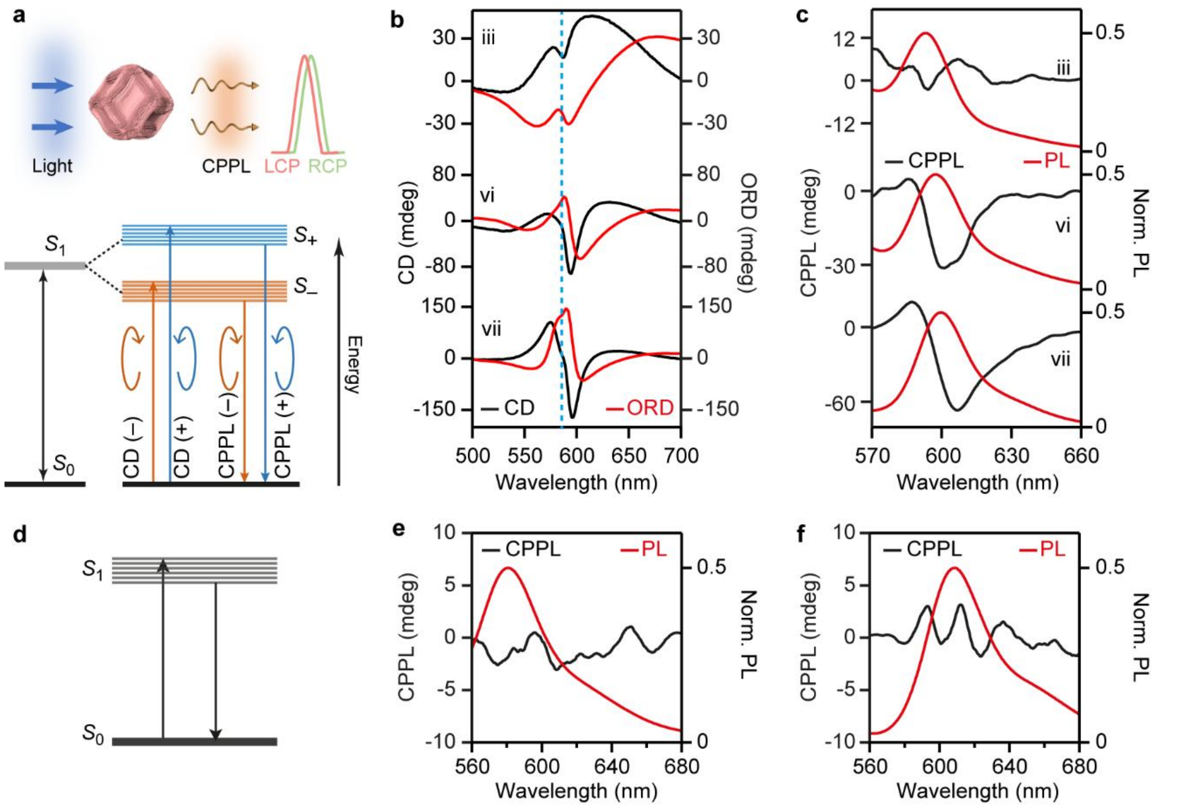

Supplementary Fig. 13 | Molecule concentration and type-dependent formation of chiral Frenkel excitons in the (432 helicoid IV NP)–molecule hybrid films. a Schematic showing the CPPL emission based on chiral excitons and the energy diagram of chiral excitons in TDBC J-aggregates. **b** Extinction CD and corresponding derived ORD spectra of the (D-432 helicoid IV NP)–(TDBC aggregate) hybrids shown in Fig. 3a in the main text. The hybrid film iii, vi, and vii were prepared at different initial TDBC monomer concentrations. The blue dashed line shows the extinction band of the hybrids. **c** Corresponding circularly polarized photoluminescence (CPPL) spectra and PL spectra measured for the hybrid films in (**b**). **d** Schematic showing the energy diagram of Rhodamine molecules, where chiral excitons are absent. **e**,**f** CPPL and PL spectra of the (D-432 helicoid IV NP)–R590 (**e**) and (D-432 helicoid IV NP)–R640 (**f**) hybrids, respectively. The CPPL responses of the (432 helicoid IV NP)–(TDBC aggregate) hybrid films are more than one order of magnitude larger than those of the hybrids containing R590 and R640.

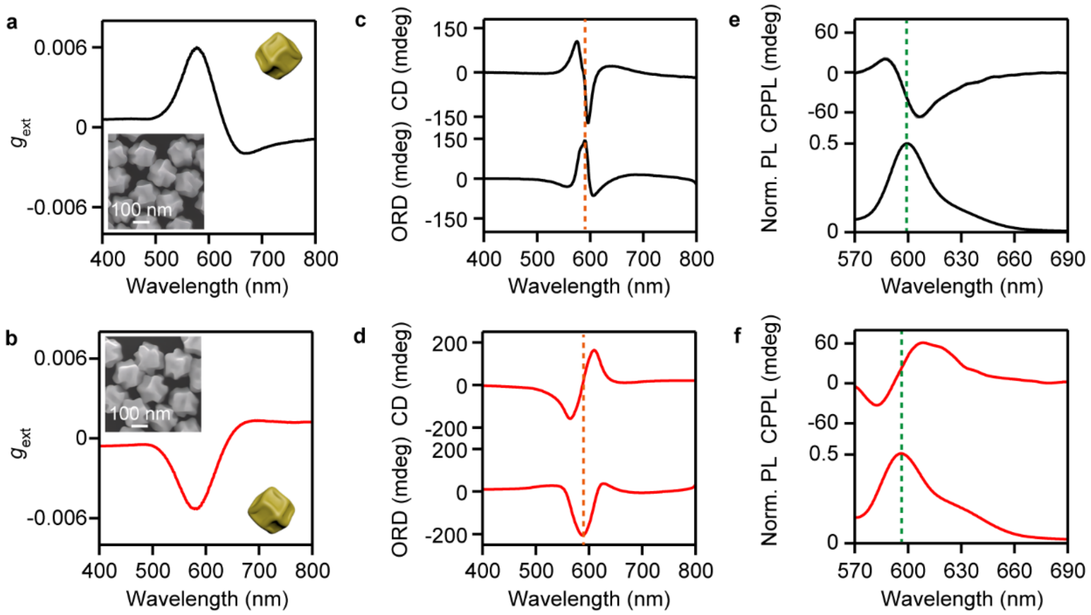

Supplementary Fig. 14 | NP geometric chirality and morphology-dependent supramolecular chirality of the formed TDBC aggregates on the NP surface. a,**b** Mirror-symmetrical extinction dissymmetry g_{ext} spectra of the D-432 helicoid IV sample and its L-type counterpart with the size D = 150 nm. **c**,**d** Extinction CD and ORD spectra of the hybrid films constructed from the D- and L-432 helicoid IV NPs. The orange dashed lines show the extinction band of the hybrids. **e**,**f** CPPL and PL spectra of the hybrid films constructed from the D- and L-432 helicoid IV NPs. The green dashed lines show the PL emission band of the hybrids. The 432-helicoid IV NPs show a very weak chiroptical extinction response. The (432 helicoid IV NP)–(TDBC aggregate) hybrid films show distinct responses from chiral excitons, indicating the chiral stacking of TDBC aggregates on the NP surface. The supramolecular chirality of TDBC aggregates depends on the structural chirality of the chiral NPs.

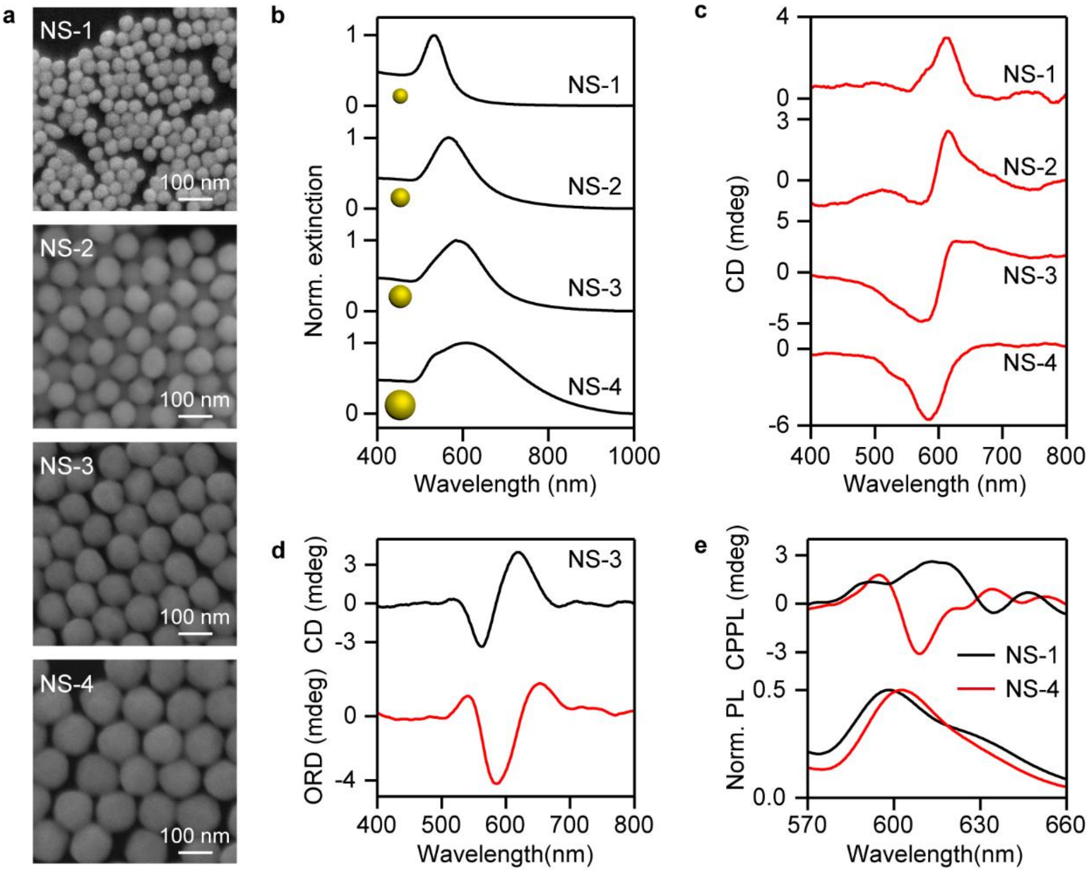

Supplementary Fig. 15 | Extinction CD and CPPL characterization of (Au nanosphere)–(TDBC aggregate) hybrid films. a,**b** SEM images and extinction spectra of Au nanosphere samples with varying diameters. **c** Extinction CD spectra of the (Au nanosphere)–(TDBC aggregate) hybrid films constructed from the differently sized Au nanospheres. **d** CD and ORD spectra of the hybrid film made of TDBC aggregates and the Au nanospheres with diameters of 100 nm and the extinction peak at 585 nm. **e** CPPL and PL spectra of the hybrid films made of TDBC aggregates and the Au nanospheres with their diameters of 80 nm (NS-1) and 120 nm (NS-4), respectively. (**d**) and (**e**) demonstrate the existence of chiral Frenkel excitons. However, the (Au nanosphere)–(TDBC aggregate) hybrids exhibit very weak extinction CD, with the ORD peak intensities being one to two orders of magnitudes smaller than those of the (432 helicoid IV NP)–(TDBC aggregate) hybrids. The (Au nanosphere)–(TDBC aggregate) hybrid films also present chiral emissions of Frenkel excitons with the CPPL responses more than 10 times smaller than those of the (432 helicoid IV NP)–(TDBC aggregate) hybrid films. These results exclude the possibility that the plasmonic near-field alone can enhance the chiroptical response of TDBC aggregates considerably to exhibit strong chiral excitonic responses, evidencing the generation of chiral excitons by the chiral stacking of TDBC aggregates on the chiral NP surface.

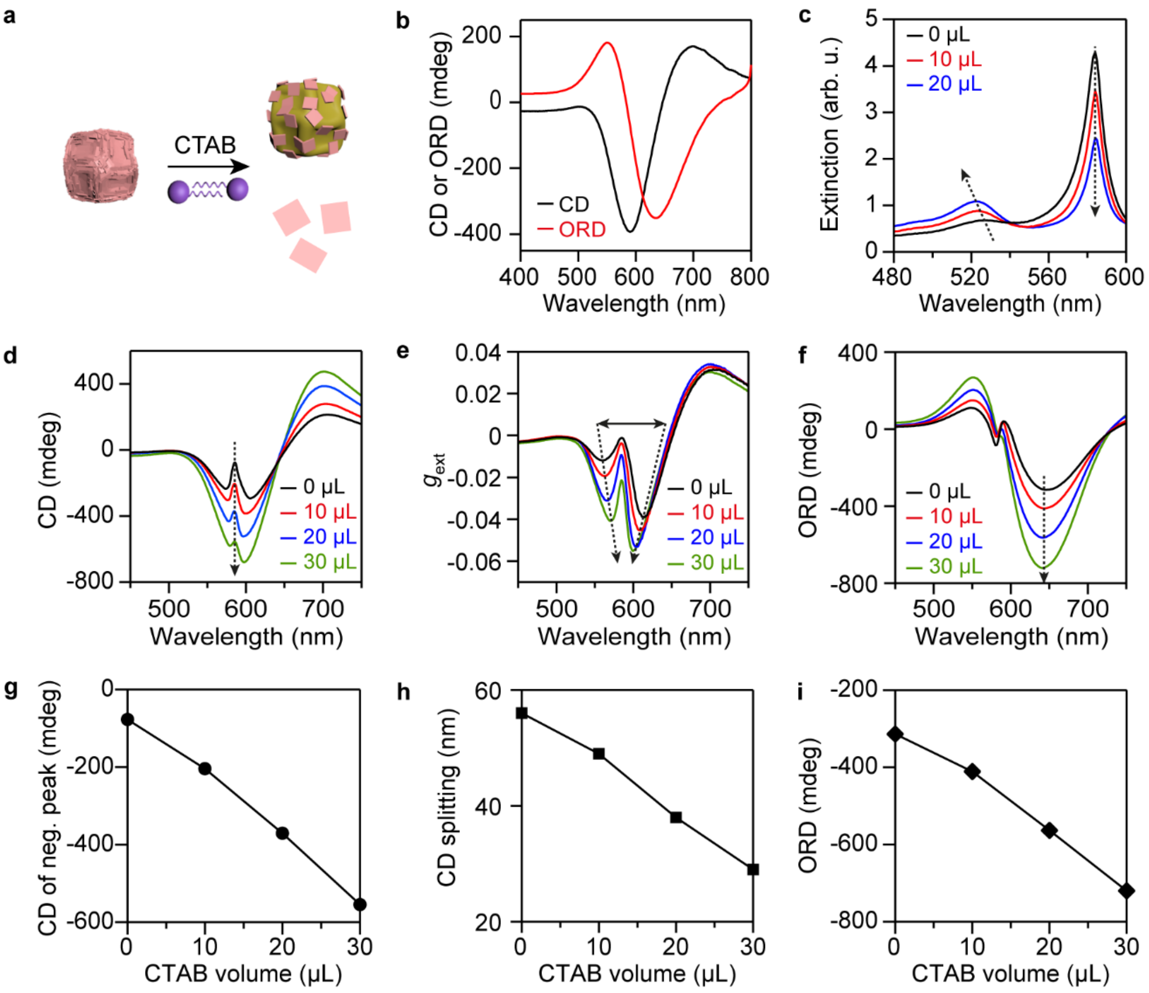

Supplementary Fig. 16 | Molecular sensing based on chiral plasmon–exciton coupling. a Schematic illustrating the sensing mechanism. The addition of specific molecules such as CTAB can trigger the disassembly of TDBC aggregates on the chiral NPs, resulting in the variation of CD spectral splitting. **b** Extinction CD and ORD spectra of the L-432 helicoid III NPs employed in our sensing experiment. **c** Change of the extinction spectra of the (L-432 helicoid III NP)–(TDBC aggregate) hybrid in a solution after the addition of CTAB (0.1 M) with increasing volumes. The intensity of the extinction peak at the TDBC J-aggregate absorption band was reduced, while the extinction peak at the TDBC monomer absorption band was increased, confirming the disassembly of TDBC aggregates on the surface of the chiral NPs. The black arrows show the changes of extinction peak at the TDBC monomer and aggregates. **d**–**f** Extinction CD spectra (**d**), g_{ext} spectra (**e**), and ORD spectra (**f**) of the (L-432 helicoid III NP)–(TDBC aggregate) hybrid in a solution after the addition of CTAB at increased volumes. The black arrows show the changes of the spectra. **g**–**i** Dependence of the peak intensity in the extinction CD spectra (**g**), energy splitting in the g_{ext} spectra (**h**), and peak intensity in the ORD spectra (**i**) on the CTAB volume.

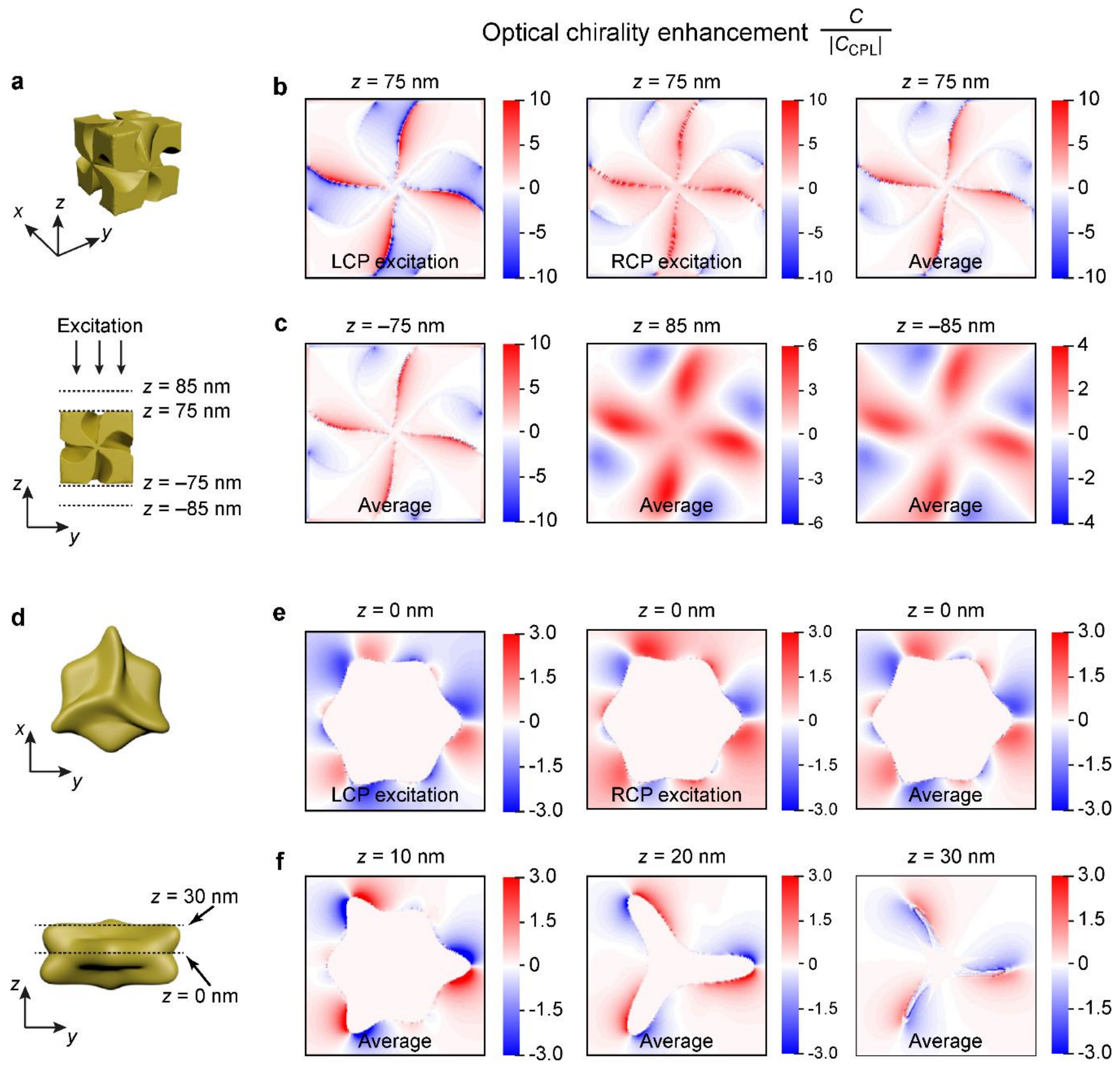

Supplementary Fig. 17 | FDTD-simulated optical chirality enhancement $C/|C_{CPL}|$ of the chiral Au 432 helicoid III NP and nanotriskelion. a Schematic showing the 432 helicoid III NP. **b** Calculated distributions of $C/|C_{CPL}|$ at the top surface of the 432 helicoid NP under LCP and RCP excitation, as well as their average. **c** Calculated distributions of average $C/|C_{CPL}|$ on the surfaces at different heights, as the bottom part in (**a**) shows. **d** Schematic showing the Au nanotriskelion. **e** Calculated distributions of $C/|C_{CPL}|$ at the center surface of the nanotriskelion under LCP and RCP excitation, as well as their average. **f** Calculated distributions of average $C/|C_{CPL}|$ on the surfaces at different heights, as the bottom part in (**d**) shows.

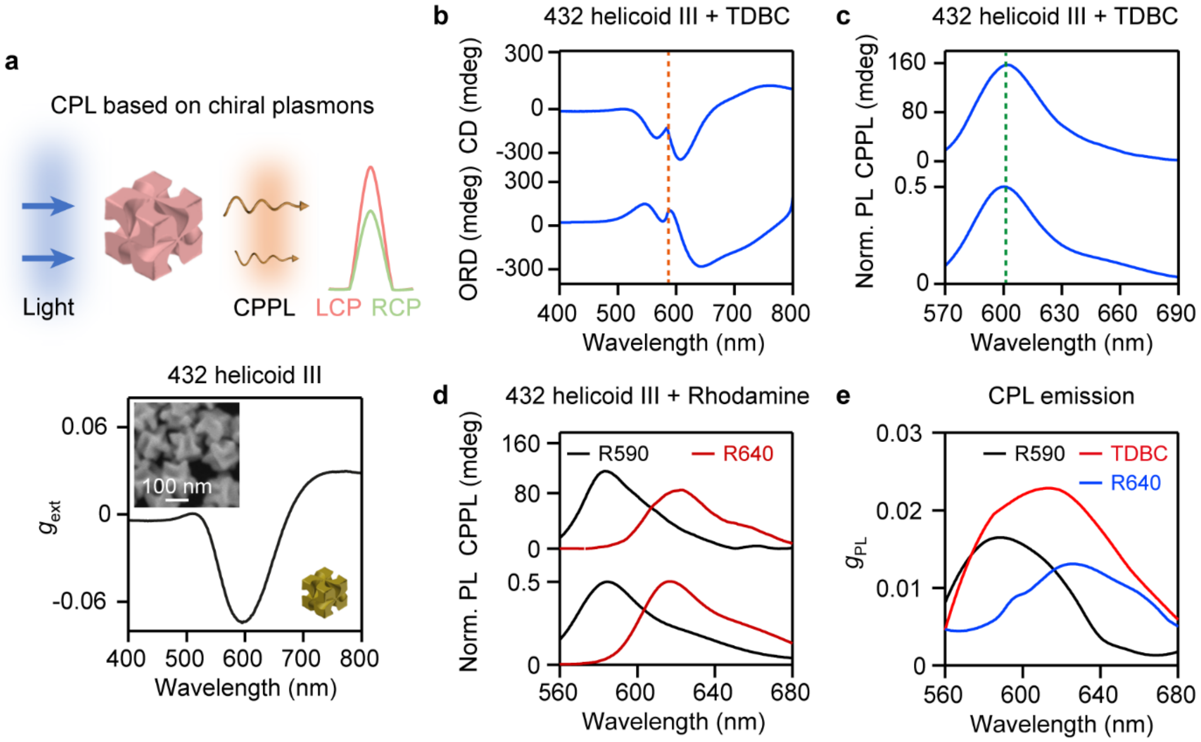

Supplementary Fig. 18 | CPPL emissions based on chiral plasmons. a Schematic illustrating the transfer of optical chirality in the 432 helicoid III–molecule hybrids (top). The L-432 helicoid III NPs with a g_{ext}-factor of −0.08 were used as the example. **b** Corresponding CD spectra of the (L-432 helicoid III NP)–(TDBC aggregate) hybrid film. The orange dashed line shows the extinction band of the hybrids. **c** CPPL spectra (right) of the (L-432 helicoid III NP)–(TDBC aggregate) hybrid film. The green dashed line shows the PL emission band of the hybrids. Compared with the (432 helicoid IV NP)–(TDBC aggregate) hybrid as shown in Supplementary Fig. 14, the (432 helicoid III NP)–(TDBC aggregate) hybrid presents degraded chiral excitonic properties, possibly because the sharp corners and edges of the 432 helicoid III NPs hinder the formation of large-area chiral J-aggregates on the NP surface. **d** CPPL spectra of the (L-432 helicoid III NP)–Rhodamine hybrid film. Compared with the (432 helicoid IV NP)–Rhodamine hybrid as shown in Supplementary Fig. 13, the (432 helicoid III NP)–Rhodamine hybrid presents strong CPPL response, demonstrating that the CPPL emissions are produced by chiral plasmons. **e** CPPL dissymmetry factor (g_{PL}) spectra of the hybrid films constructed from the 432 helicoid III NPs and R590, TDBC, and R640, respectively. The 432 helicoid III NPs with g_{ext} = −0.08 at 590 nm resulted in the hybrid films showing fluorescent g_{PL} = +0.017 at 582 nm, +0.023 at 610 nm, +0.013 at 620 nm when the NPs were hybridized with R590, TDBC aggregate, and R640, respectively.

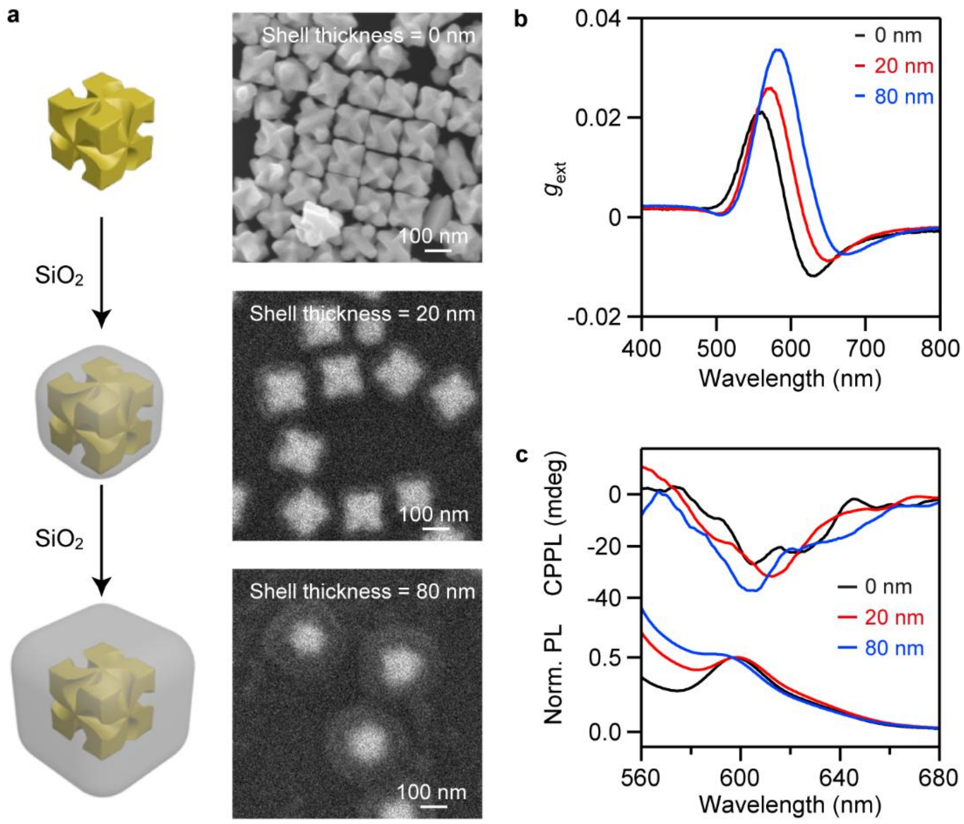

Supplementary Fig. 19 | CPPL emissions of the hybrid films constructed from the silica-coated D-432 helicoid III NPs and TDBC aggregates. a Schematic illustrating the preparation of the D-432 helicoid III NPs coated with silica shell (left) and SEM images of the prepared core@shell structures with different SiO_2 shell thicknesses (right). **b** Extinction dissymmetry factor (g_{ext}) spectra of the D-432 helicoid III NPs coated with silica shell of varying thicknesses. **c** CPPL and PL spectra of the hybrid films of the silica-coated D-432 helicoid III NPs and TDBC aggregates.

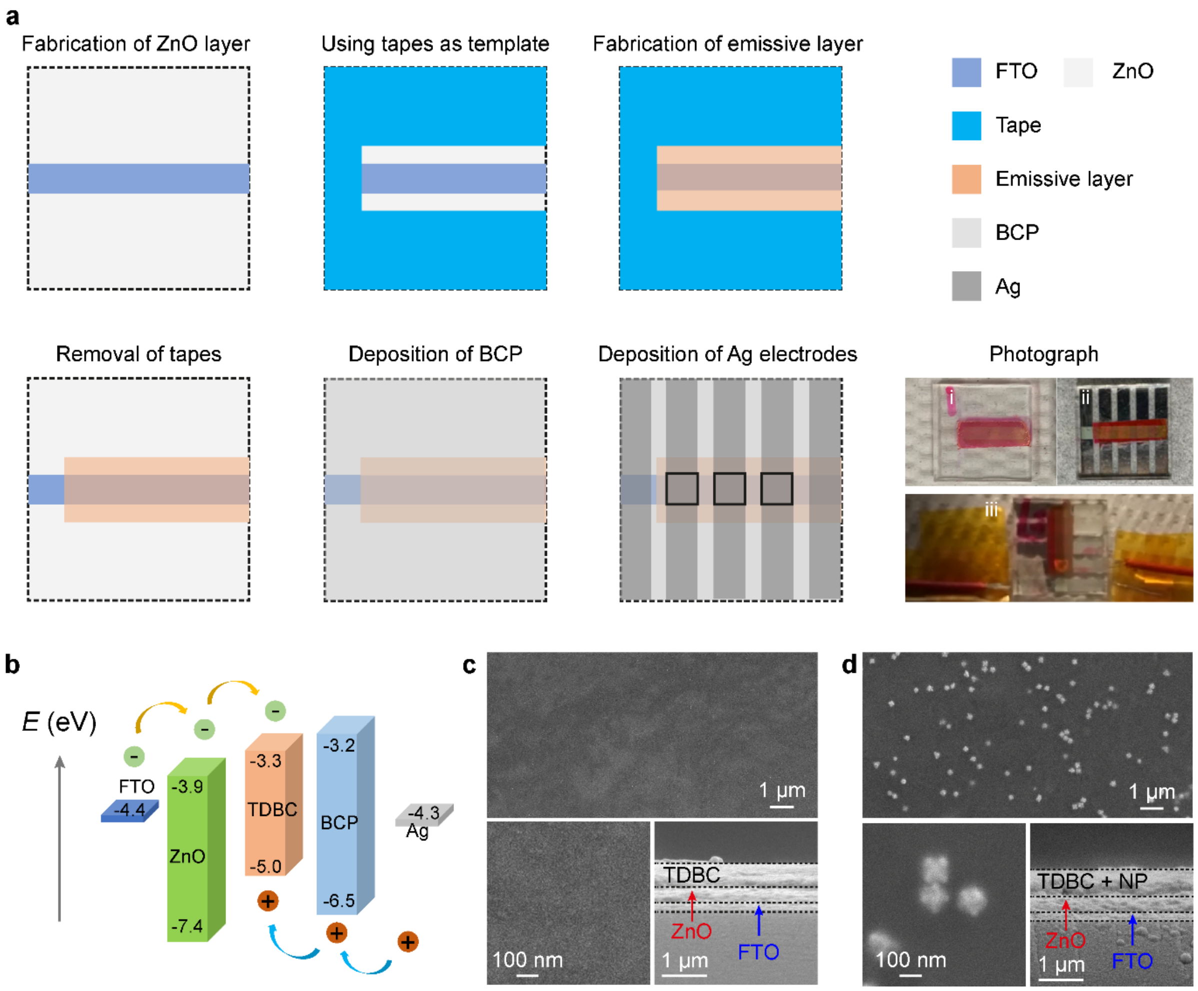

Supplementary Fig. 20 | Fabrication and characterization of the OLED devices. a Schematics illustrating the preparation process of the OLED devices. The photographs are also provided (bottom right corner) to show a typical OLED device at the different preparation stages, including (i) the structure of FTO/ZnO/emissive layer/BCP, (ii) the structure of FTO/ZnO/emissive layer/BCP/Ag, and (iii) the OLED device after connection to the circuit. **b** Energy level diagram of the OLED using TDBC aggregates as the emissive layer[3]. ZnO and BCP were selected as the electron and hole transport layer, respectively. **c** SEM images of the emissive layer in the L-OLED-2 device, including the image of the (L-432 helicoid III NP)–(TDBC aggregate) hybrid film surface (top), the magnified SEM images demonstrating that the L-432 helicoid III NPs were embedded in the TDBC aggregates (bottom, left), the cross-section image of the L-OLED-2 device before the deposition of BCP and Ag, revealing the FTO, ZnO, and emissive layer (bottom, right). **d** SEM images of the emissive layer in the Ref-OLED device, including the image of the TDBC aggregate hybrid film surface (top), the magnified SEM images demonstrating the high uniformity of the TDBC film (bottom, left), the cross-section image of the Ref-OLED device before the deposition of BCP and Ag, revealing the FTO, ZnO, and emissive layer (bottom, right). The thickness of the TDBC layer in Ref-OLED is 560 ± 37 nm. The (432 helicoid III NP)–(TDBC aggregate) hybrid film in CP-OLED-2 showed a larger thickness of 640 ± 50 nm because of the large size of the 432 helicoid III NPs.

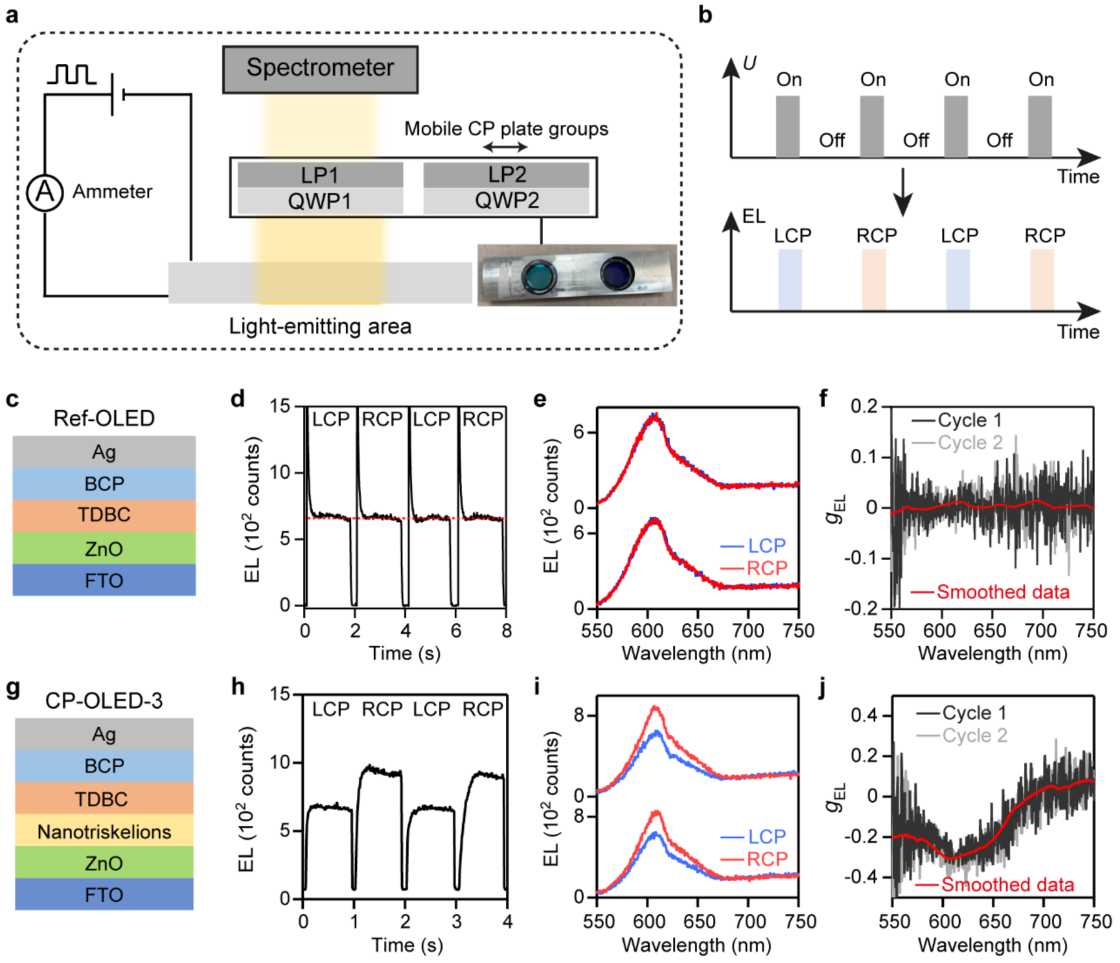

Supplementary Fig. 21 | Detailed methodologies for CPEL measurements. a Schematic of the home-built optical setup for CPEL measurements. Two sets of circular polarized plates were employed. Each was composed of a quarter-wave plate (QWP) and an ultrathin linear polarizer (LP). These two plate sets were integrated into one bracket (see the photograph), one for measuring the LCP component and the other for recording the RCP component of the emission. **b** Schematic of the process for the measurement of LCP and RCP components. Two voltage pulses with a 2-s interval were applied to accurately measure the intensity of CPEL components. During a 2-s interval, the plate groups were rapidly moved to switch the component measurement. Various OLED devices of the same type were examined to ensure the accuracy of the CPEL measurements. **c**–**f** CPEL performance of the Ref-OLED device. The TDBC aggregate film was used as the emissive layer (**c**). The transient profiles of the EL intensity were recorded when the LCP and RCP components of the EL were selected (**d**). Unprecedented overshoot spikes of the EL turn-on transience appeared when a voltage of U = 6 V was applied. The CPEL spectra were obtained in two cycles (**e**). We therefore calculated the electroluminescent g_{EL} spectra (**f**), demonstrating the achiral EL from the Ref-OLED device. The TDBC J-aggregate film shows a very weak chiroptical response and the Ref-OLEDs exhibited a nearly achiral EL feature. **g**–**j** CPEL performance of the CP-OLED-3 device. A combination of the TDBC aggregate film and L-nanotriskelions was used as the emissive layer (**g**). The transient profiles of the LCP and RCP EL intensity were recorded (**h**). The CPEL spectra were obtained in two cycles (**i**). We calculated the electroluminescent g_{EL} spectra (**j**), demonstrating the chiral EL emission from the CP-OLED-3 device. The CP-OLED-3 device displayed distinctly different LCP and RCP EL intensities.

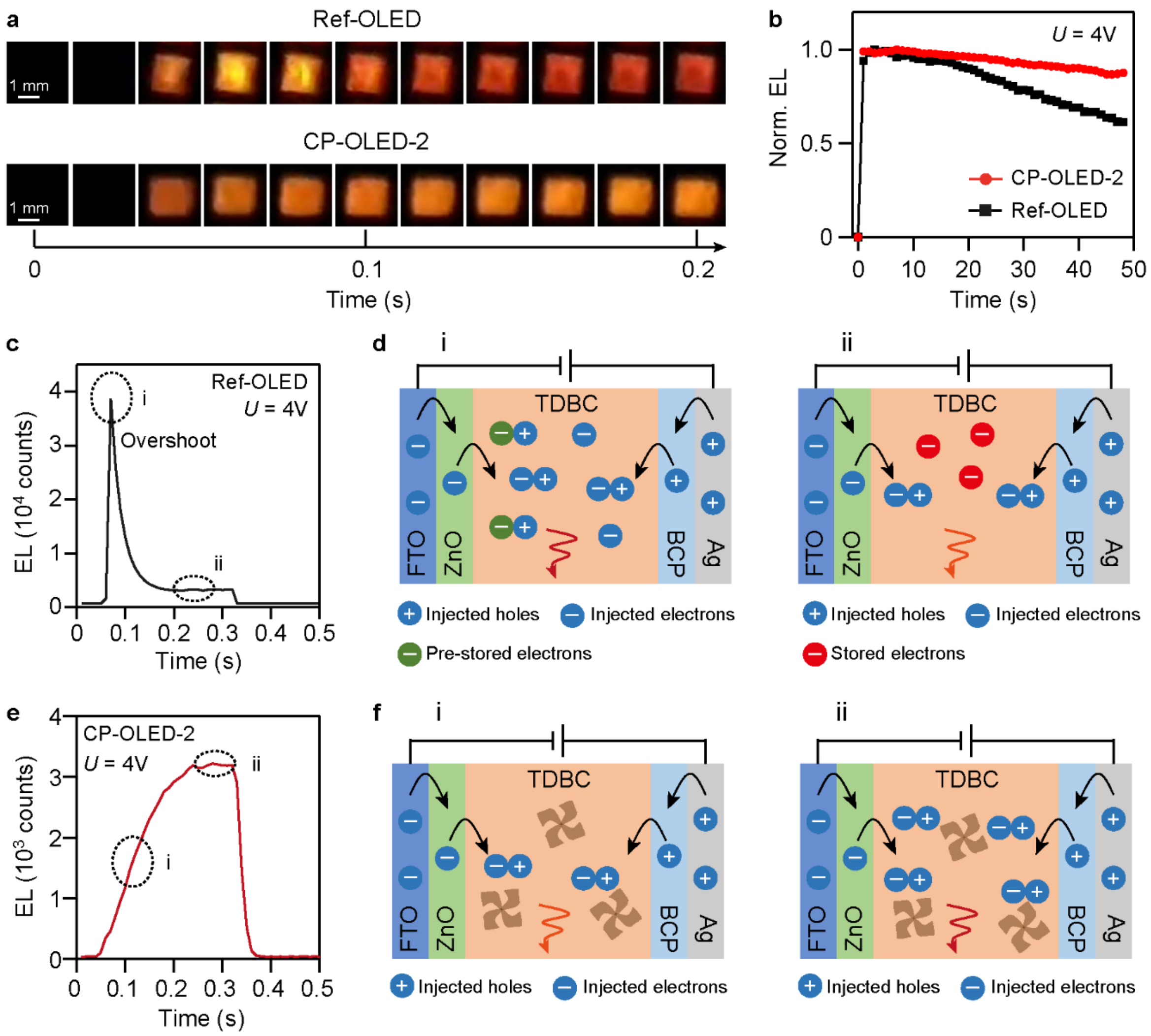

Supplementary Fig. 22 | Comparison of the Ref-OLED and CP-OLED-2 devices in terms of the overshoot effect. a Time-dependent luminating photographs of the OLED devices after a voltage of *U* = 6 V was applied. **b** Normalized EL intensity changes over time for Ref-OLED and CP-OLED-2. The CP-OLED-2 device exhibits a higher stability because of the suppression of the overshoot effect. **c** Transient EL profiles of a Ref-OLED device. **d** Schematic illustrating the overshoot phenomenon in the Ref-OLED devices. The luminance first increases and overshoots to a maximum value (i) before decreasing to a steady value (ii). TDBC monomers have two imidazole rings and show electron-donating properties. In the Ref-OLED device, after a few rounds of electrical switching, some electrons were pre-stored in the TDBC aggregates. The new round of electrical switching triggers the radiative recombination of the injected holes and the pre-stored electrons (i), producing a spike with a super-high intensity at the transient EL rising edge[81]. After the pre-stored electrons are consumed, the luminance decreases until it reaches a steady value, and new electrons start to accumulate in the TDBC aggregates again (ii). A recovery time of 0.1–0.2 s is necessary for the EL of Ref-OLED to reach its steady state. The overshoot effect can be observed in the CP-OLED-3 devices. **e** Transient EL profile of a CP-OLED-2 device. **f** Schematic illustrating the suppression of the overshoot effect. The CP-OLED-1 and CP-OLED-2 devices used the hybrids of TDBC aggregates and the chiral Au NPs with excellent electrical conductivity. The amount of pre-stored electrons can be significantly reduced, thereby eliminating the overshoot effect. The measured CP-OLED-2 device underwent a relatively slow rise in its luminance intensity upon the application of a voltage, which took about 0.2 s until the

luminance intensity reached a steady state. The slower rise of the luminance intensity in the CP-OLED-2 devices results from both the lack of pre-stored carriers and the large emissive layer thickness of the CP-OLED-2 devices (640 ± 50 nm). After the application of a voltage pulse, the transport and recombination of charge carriers requires longer time.

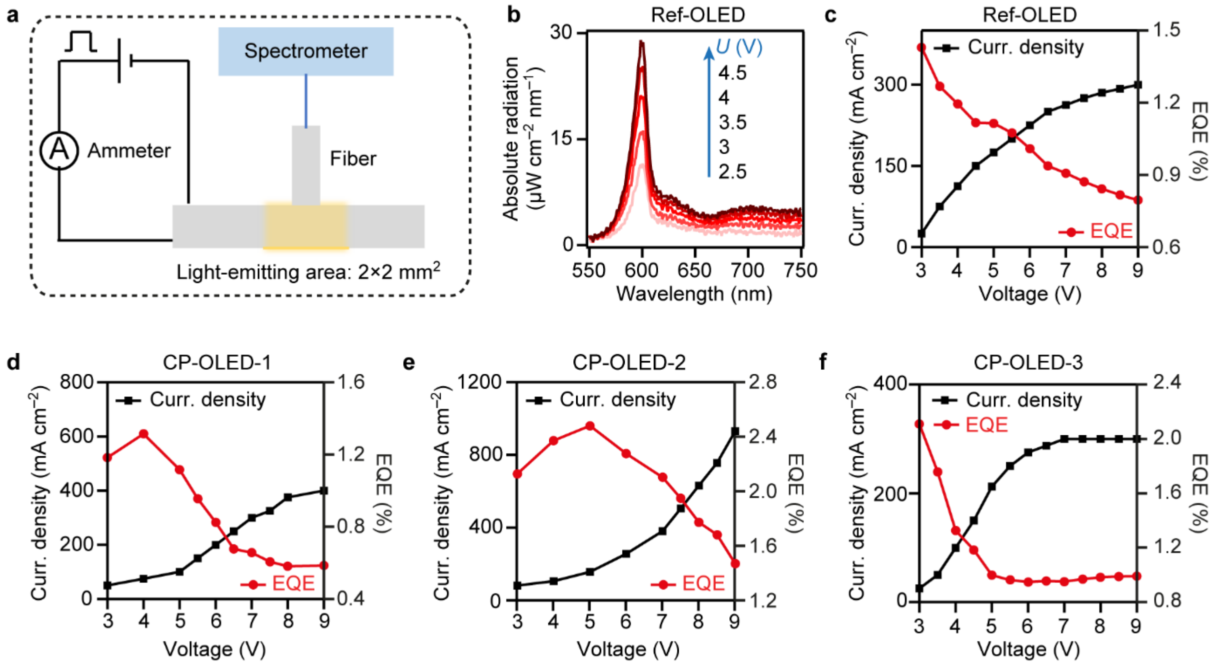

Supplementary Fig. 23 | EQE measurements of the OLED devices. a Schematic showing the optical setup for the measurement of the external quantum efficiency (EQE) of an OLED device. The emitted EL light was collected by an optical fiber and directed to a spectrometer. The current was simultaneously recorded from the ammeter under the application of different voltages. **b** Absolute radiation spectra at varying applied voltages for a typical OLED device. **c**–**f** Voltage-dependent current density and EQE for a Ref-OLED device (**c**), a CP-OLED-1 device (**d**), a CP-OLED-2 device (**e**), and a CP-OLED-3 device (**f**), respectively.

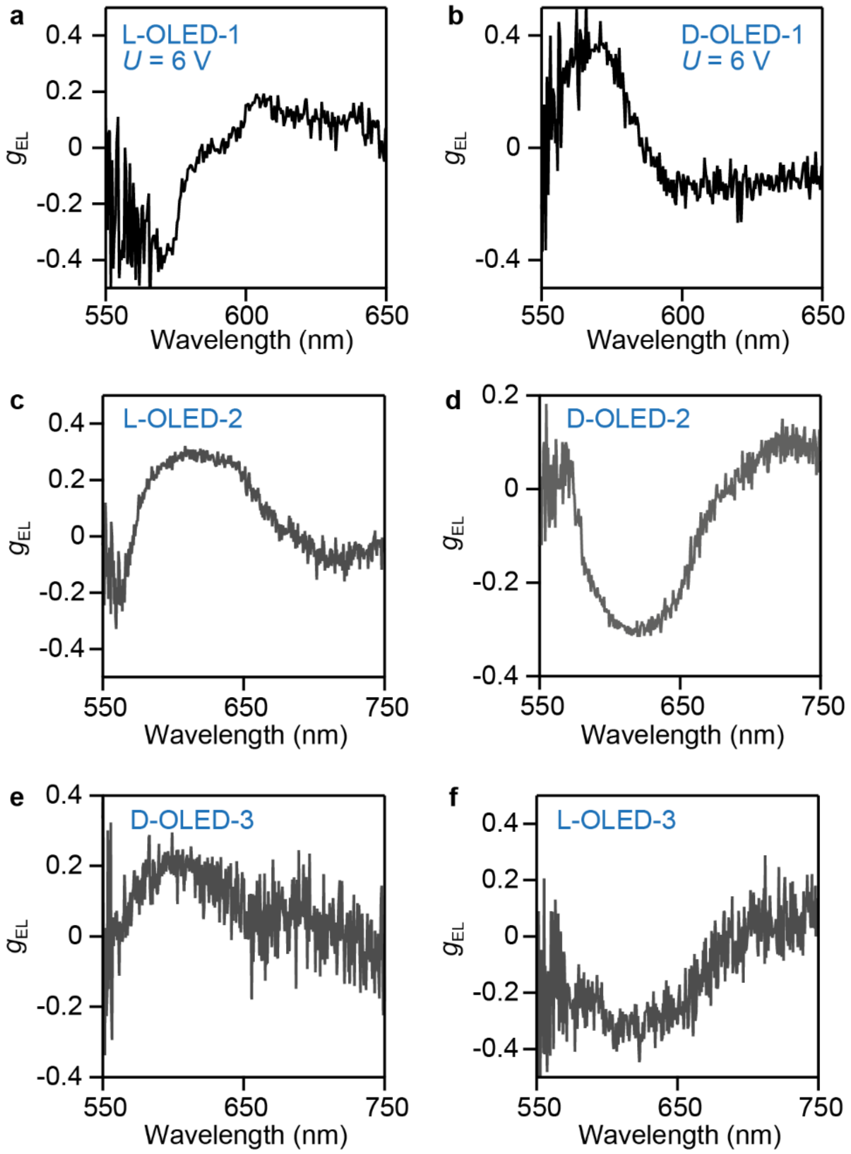

Supplementary Fig. 24 | Originally measured electroluminescent g_{EL} spectra. a,**b** L-OLED-1 (**a**) and D-OLED-1 (**b**) devices under the applied voltage of U = 6 V. The corresponding smoothed g_{EL} spectra are shown in Fig. 4b in the main text. **c**,**d** L-OLED-2 (**c**) and D-OLED-2 (**d**) devices working at U = 9 V. The corresponding smoothed g_{EL} spectra are shown in Fig. 4e in the main text. **e**,**f** D-OLED-3 (**e**) and L-OLED-3 (**f**) devices working at U = 5 V. The corresponding smoothed g_{EL} spectra are shown in Fig. 4h in the main text.

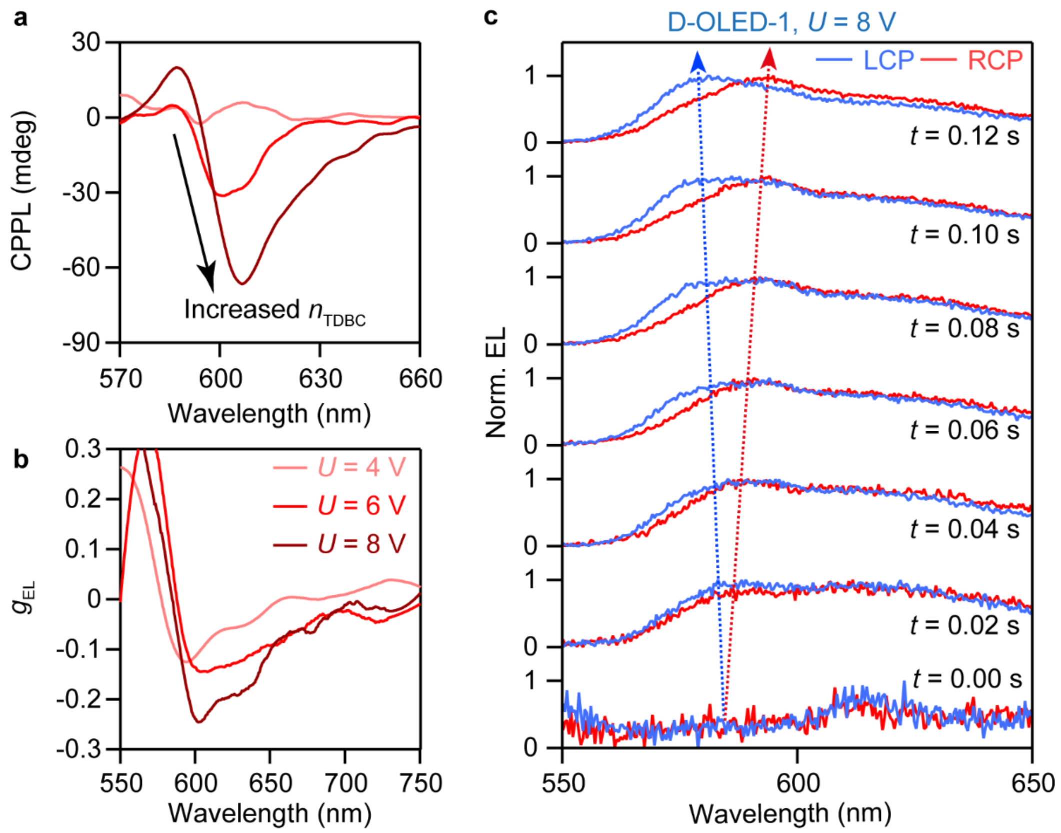

Supplementary Fig. 25 | Comparison between CPPL and CPEL measurements on the (432 helicoid IV NP)–(TDBC aggregate) hybrids. a CPPL measurement on the (D-432 helicoid IV NP)–(TDBC aggregate) hybrids. The increased TDBC amount caused the gradual enrichment of chiral excitons, leading to the increased CPPL intensity. **b** CPEL measurement on the CP-OLED-1 device employing the (D-432 helicoid III NP)–(TDBC aggregate) hybrid film as the emissive layer. The comparison among the CPEL measurements on the same device working at different voltages suggests that as the applied voltage *U* is enlarged, excited chiral excitons near the NP surface become enriched while the EL from achiral excitons reaches saturation, giving rise to superior CPEL of the OLED device working at high voltages. **c** Transient CPEL profiles of the D-OLED-1device after the 8 V voltage was applied to the device. The CPEL spectra were obtained at an exposure time of 0.01 s. The CPEL intensity increased from ≈ 100 counts to ≈ 500 counts, with a gradually enlarged splitting between the LCP and RCP EL peaks. Such results indicate that the increase of injected electron–hole pairs can guide the generation of chiral excitons with increased numbers.

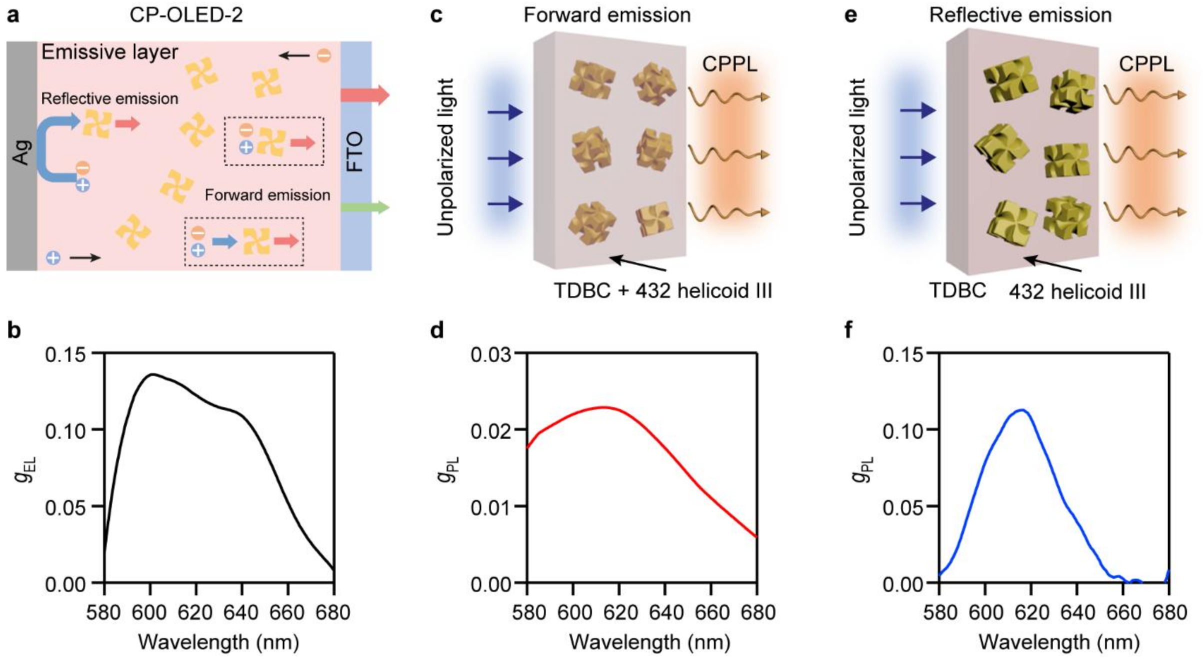

Supplementary Fig. 26 | Comparison between CPPL and CPEL measurements on the (432 helicoid III NP)–(TDBC aggregate) hybrids. a Schematic illustrating the CPEL emissions in the CP-OLED-2 device. Previous works have demonstrated that the EL emits forward to the transparent FTO electrode or emits backward and gets reflected by the Ag back electrode[82]. Both the forward and backward-reflected emissions can be circularly polarized by the chiral NPs to carry the same handedness, preventing any circular polarization cancellation and significantly boosting the asymmetric EL. **b** The CP-OLED-2 device using the L-432 helicoid III NPs with g_{ext} of −0.08 at 600 nm exhibits g_{EL} = +0.13. The SEM image and g_{ext} spectrum of the L-432 helicoid III NPs are shown in Supplementary Fig. 28a,b. **c**,**d** CPPL emission in the (432 helicoid III NP)–(TDBC aggregate) hybrid film, mimicking the forward emissions in the CP-OLED-2 device and showing a g_{PL} = +0.022 at 600 nm. **e**,**f** CPPL emissions in the TDBC aggregate film assembled with a separate layer of L-432 helicoid III, mimicking the backward emissions in the CP-OLED-2 device and showing a g_{PL} = +0.12 at 600 nm. These experimental results confirmed that the g_{EL}-factor in CP-OLED-2 was originated from both the forward and backward-reflected emissions.

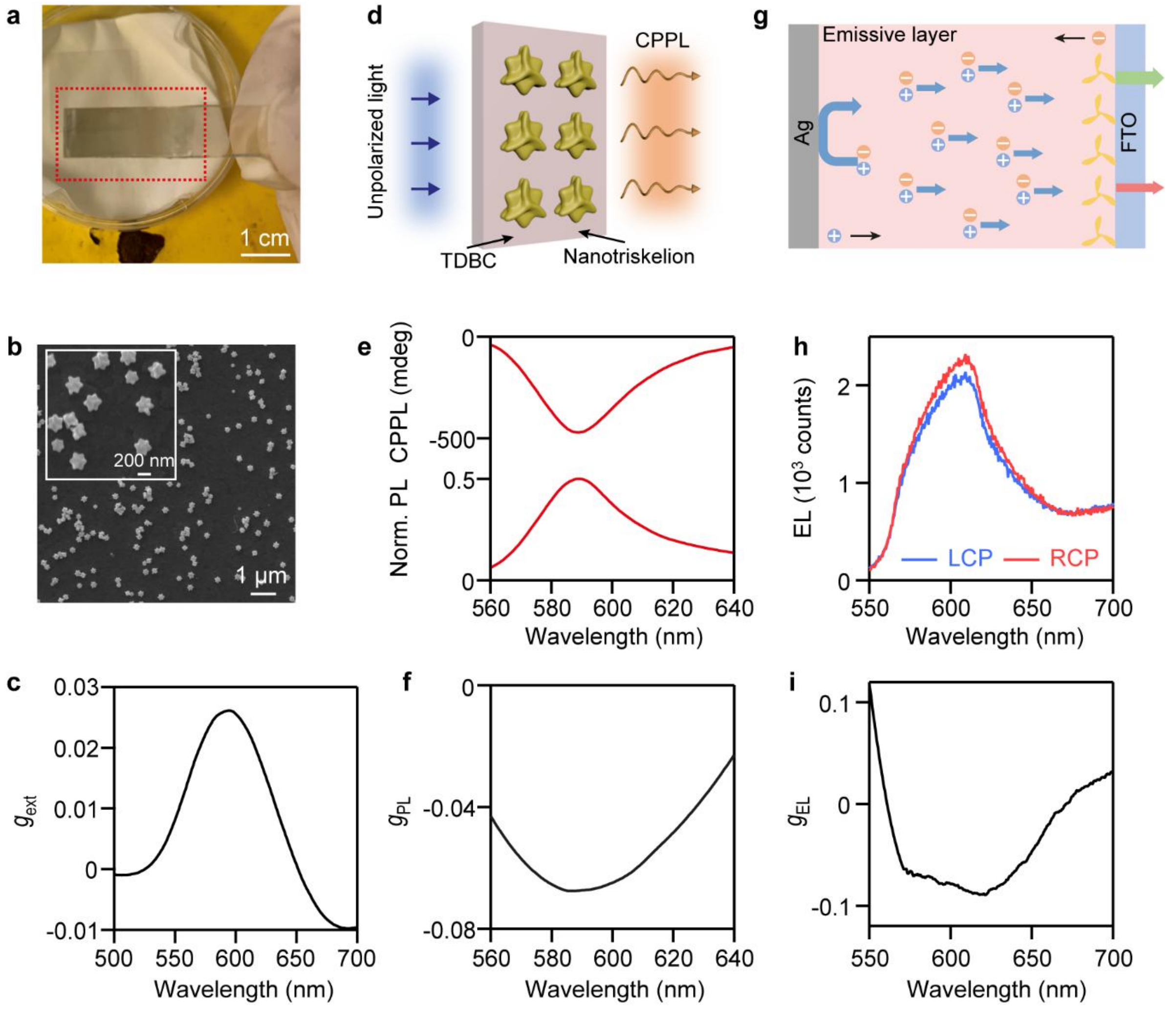

Supplementary Fig. 27 | Comparison between CPPL and CPEL measurements of TDBC films assembled with a layer of aligned chiral Au nanotriskelions. **a** Photograph of a transparent flat substrate deposited with the aligned Au nanotriskelions. **b** SEM image of the Au nanotriskelion layer in (**a**). The inset shows the magnified SEM image of the sample. The chiral NPs lie on the flat substrate and are aligned in an up-and-down direction. **c** g_{ext} spectrum of the Au nanotriskelions in (**b**). **d** Schematic illustrating the CPPL measurement of the TDBC film assembled with a layer of chiral Au nanotriskelions. **e**,**f** CPPL spectra (**e**, top), PL spectra (**e**, bottom), and g_{PL} spectra (**f**) of the TDBC film assembled with the aligned chiral Au nanotriskelion layer. **g** Schematic illustrating the CPEL emissions in the CP-OLED-3 device. The EL emits forward to the transparent FTO electrode or emits backward and gets reflected by the Ag back electrode. All the emitted unpolarized photons are circularly polarized by the nanotriskelions. **h** CPEL spectra of the CP-OLED-3 device using the same Au nanotriskelions layer in (**b**,**c**). **i** g_{EL} spectrum of the CP-OLED-3 device. The L-nanotriskelions with g_{ext} = +0.025 at 600 nm support CPPL emissions with g_{PL} = −0.07. The same L-nanotriskelions was used in the CP-OLED-3 device, leading to CPEL emissions of g_{EL} = −0.08 at the same wavelength. The similar *g*-factors demonstrated that only the plasmonic polarization-filtering effect contributes to the device CPEL.

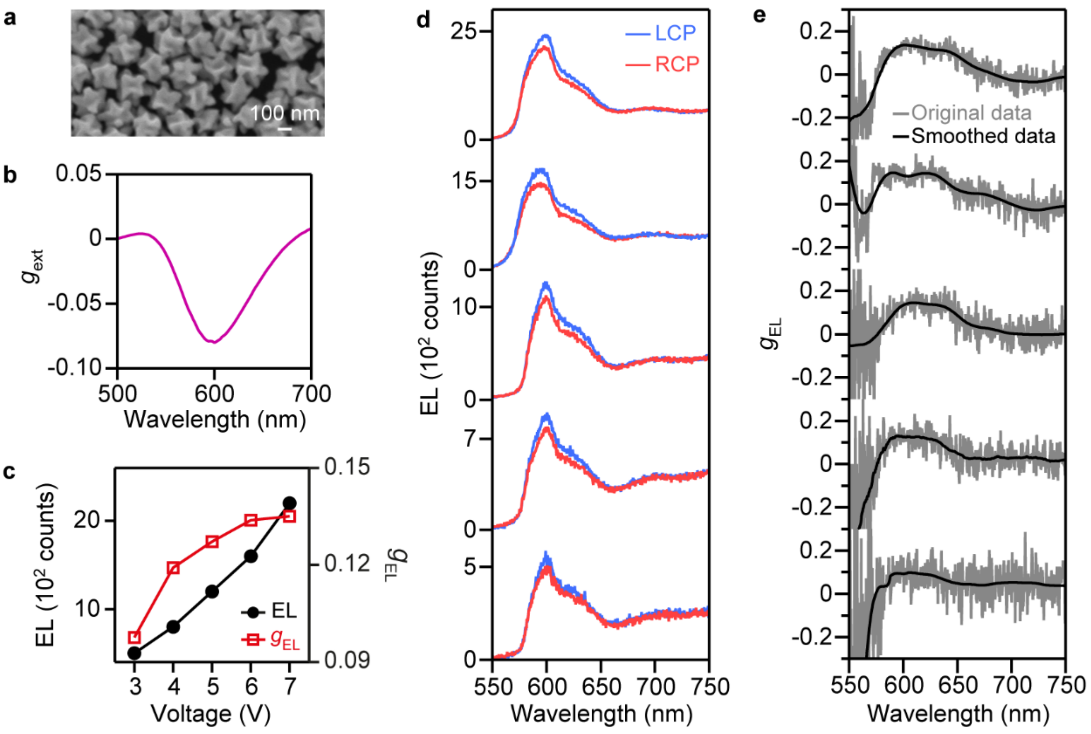

Supplementary Fig. 28 | Dependences of the EL intensity and g_{EL} on the applied voltage in a CP-OLED-2 device. The L-432 helicoid III NPs with g_{ext} of −0.08 at 600 nm were employed. **a**,**b** SEM image (**a**) and g_{ext} spectrum (**b**) of the L-432 helicoid III NPs. **c** Voltage dependences of the EL intensity and g_{EL} of the CP-OLED-2 device. As the applied voltage U was increased, the device exhibited amplified EL intensities and $|g_{EL}|$, with g_{EL} reaching a plateau at high voltages. **d**,**e** CPEL (**d**) and g_{EL} (**e**) spectra of the CP-OLED-2 device when the applied voltage U was set at the values shown in Fig. 5c in the main text. The transient EL intensities of the CP-OLED-2 device when U was swept between zero and linearly increasing values are shown in Fig. 5c in the main text.

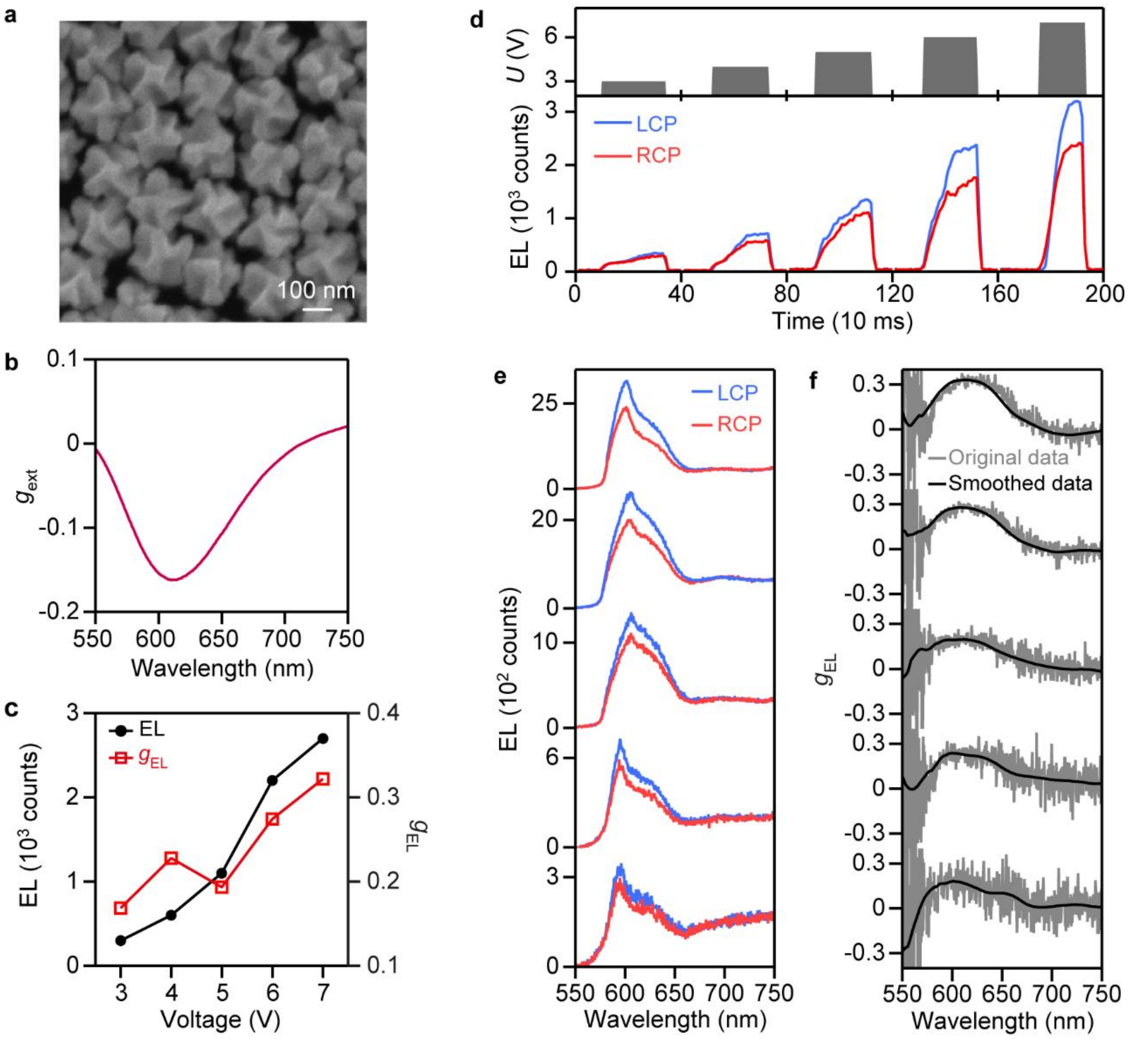

Supplementary Fig. 29 | Dependences of the EL intensity and g_{EL} on the applied voltage in a L-type CP-OLED-2 device. The L-432 helicoid III NPs with g_{ext} of −0.16 at 610 nm were employed. **a**,**b** SEM image (**a**) and g_{ext} spectrum (**b**) of the L-432 helicoid III NPs. **c** Voltage dependences of the EL intensity and g_{EL} of the CP-OLED-2 device. As the applied voltage *U* was increased, the device exhibited amplified EL intensities and $|g_{EL}|$, with the g_{EL} reaching a plateau at high voltages. **d** Transient EL intensities of the CP-OLED-2 device when *U* was swept between zero and linearly increasing values. The exposure time to collect each data point was set at 0.01 s. **e**,**f** CPEL (**e**) and g_{EL} (**f**) spectra of the CP-OLED-2 device when the applied voltage *U* was set at the values shown in (**d**).

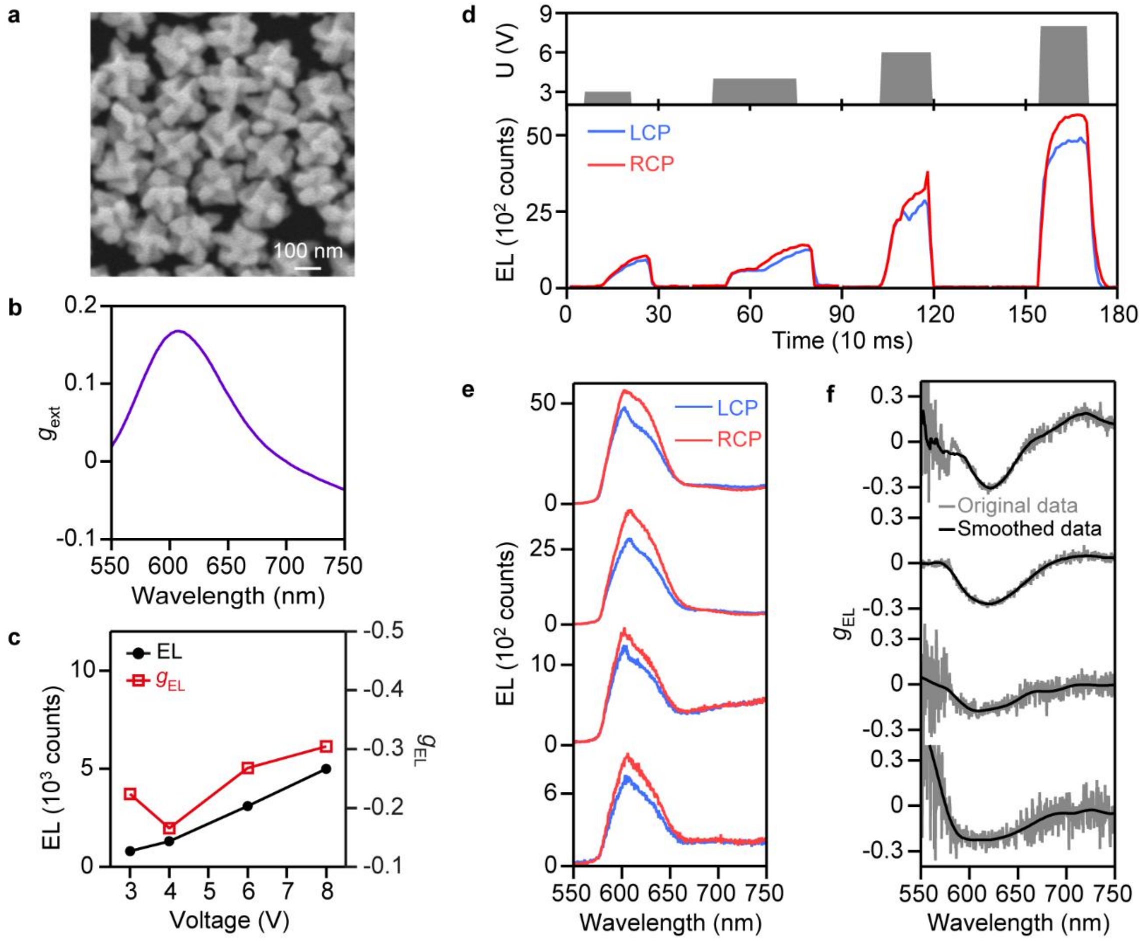

Supplementary Fig. 30 | Dependences of the EL intensity and g_{EL} on the applied voltage in a D-type CP-OLED-2 device. The D-432 helicoid III NPs with g_{ext} of +0.17 at 610 nm were employed. **a**,**b** SEM image (**a**) and g_{ext} spectrum (**b**) of the D-432 helicoid III NPs. **c** Voltage dependences of the EL intensity and g_{EL} of the CP-OLED-2 device. As the applied voltage *U* was increased, the device exhibited amplified EL intensities and $|g_{EL}|$, with the g_{EL} reaching a plateau at high voltages. **d** Transient EL intensities of the CP-OLED-2 device when *U* was swept between zero and linearly increasing values. The exposure time to collect each data point was set at 0.01 s. **e**,**f** CPEL (**e**) and g_{EL} (**f**) spectra of the CP-OLED-2 device when the applied voltage *U* was set at the values shown in (**d**).

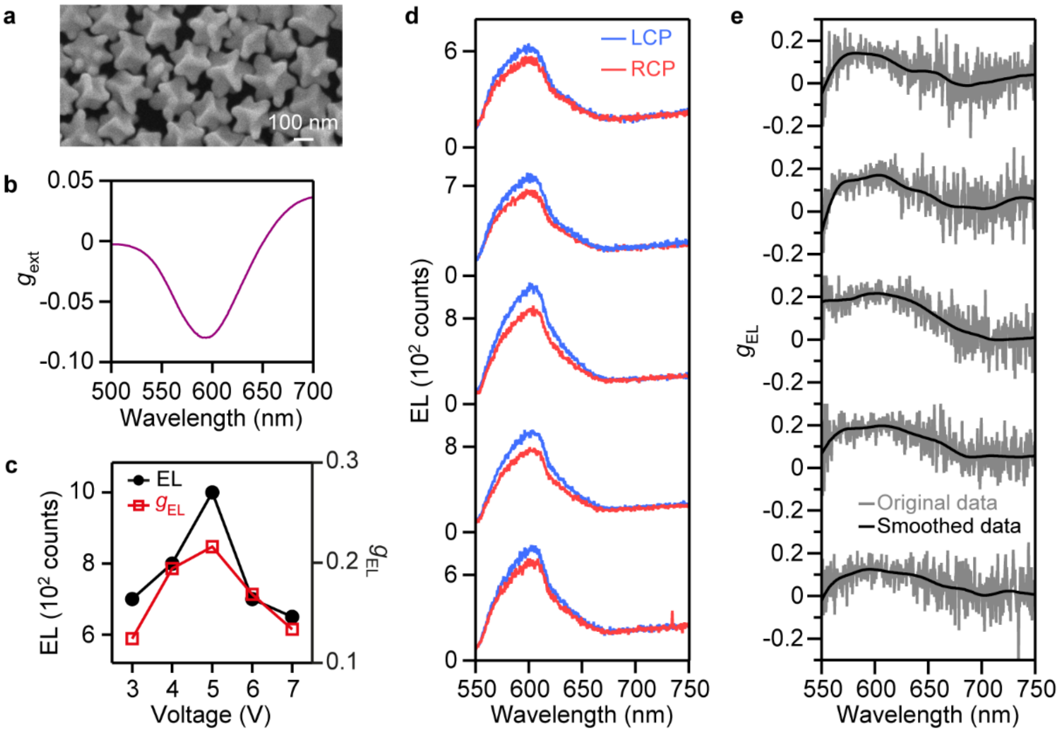

Supplementary Fig. 31 | Dependences of the EL intensity and g_{EL} on the applied voltage in a D-type CP-OLED-3 device. The D-nanotriskelions with g_{ext} of −0.08 at 600 nm were employed. **a**,**b** SEM image (**a**) and g_{ext} spectrum (**b**) of the D-nanotriskelions. **c** Voltage dependences of the EL intensity and g_{EL} of the CP-OLED-3 device. **d**,**e** CPEL (**d**) and g_{EL} (**e**) spectra of the CP-OLED-3 device when the applied voltage *U* was set at the values shown in Fig. 5d in the main text. The transient EL intensities of the CP-OLED-3 device when *U* was swept between zero and linearly increasing values are shown in Fig. 5d in the main text.

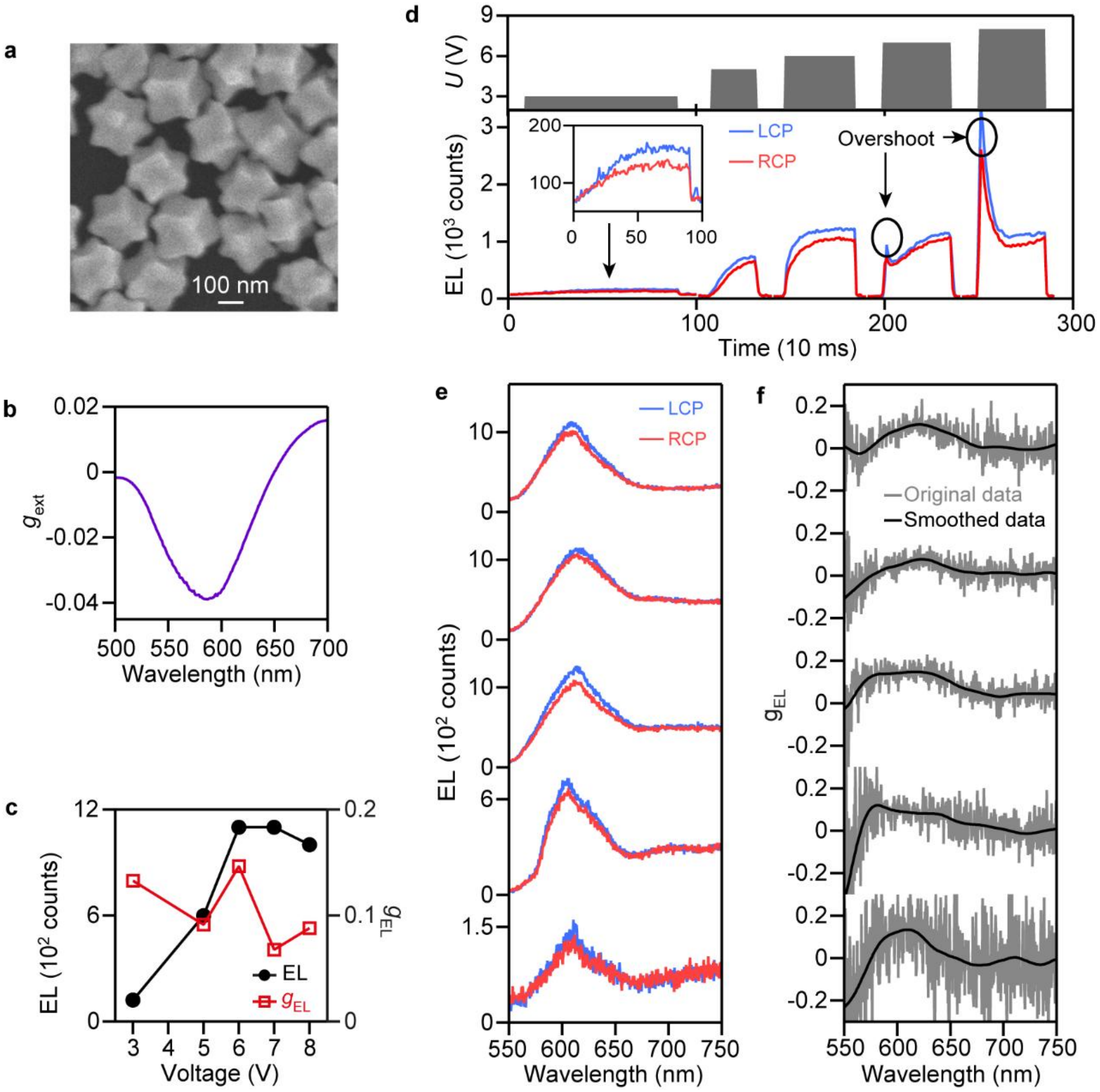

Supplementary Fig. 32 | Dependences of the EL intensity and g_{EL} on the applied voltage in a D-type CP-OLED-3 device. The D-nanotriskelions with g_{ext} of −0.04 at 585 nm were employed. **a**,**b** SEM image (**a**) and g_{ext} spectrum (**b**) of the D-nanotriskelions. **c** Voltage dependences of the EL intensity and g_{EL} of the CP-OLED-3 device. **d** Transient EL intensities of the CP-OLED-3 device when U was swept between zero and linearly increasing values. The exposure time to collect each data point was set at 0.01 s. **e**,**f** CPEL (**e**) and g_{EL} (**f**) spectra of the CP-OLED-3 device when the applied voltage U was set at the values shown in (**d**).

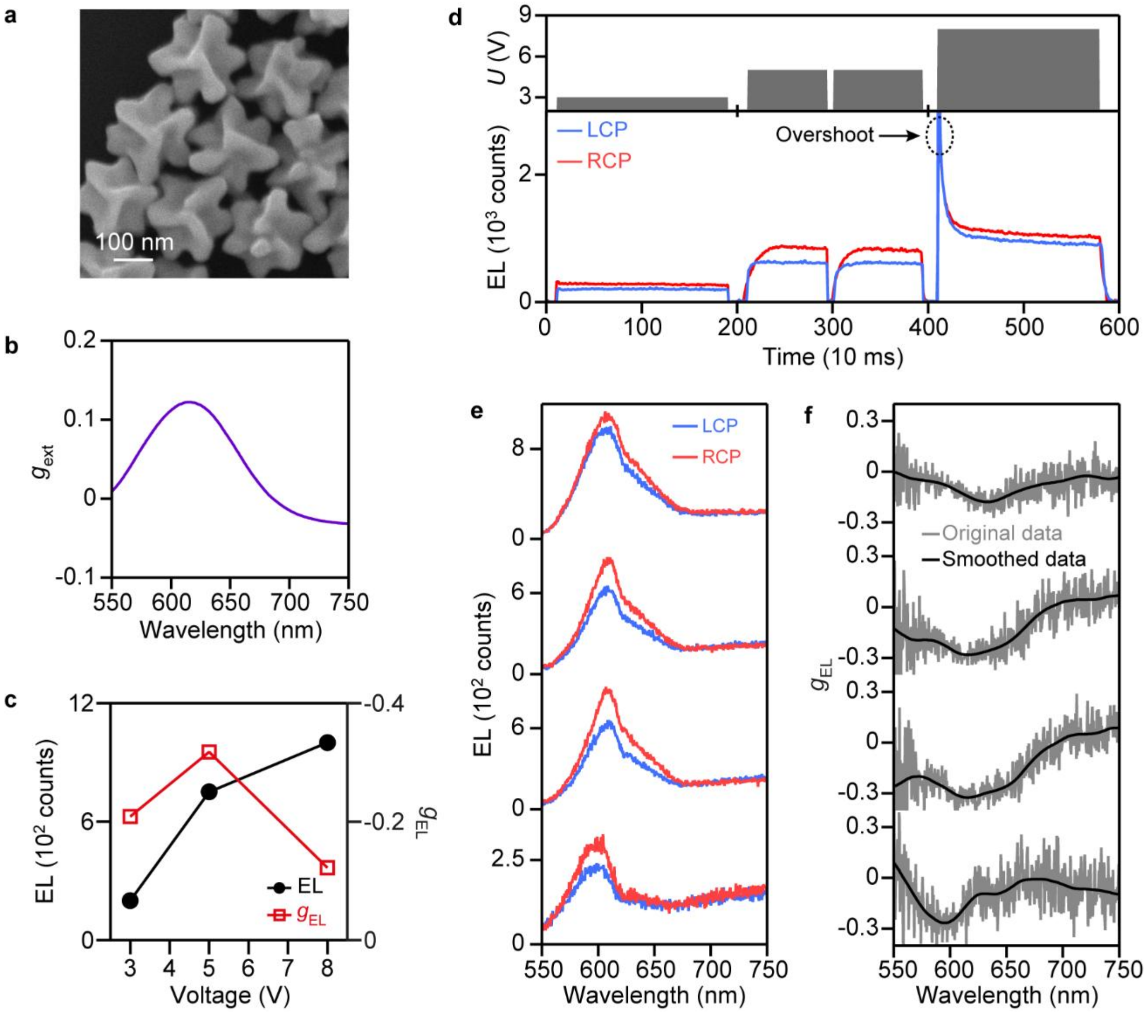

Supplementary Fig. 33 | Dependences of the EL intensity and g_{EL} on the applied voltage in a L-type CP-OLED-3 device. The L-nanotriskelions with g_{ext} of 0.12 at 610 nm were employed. **a**,**b** SEM image (**a**) and g_{ext} spectrum (**b**) of the L-nanotriskelions. **c** Voltage dependences of the EL intensity and g_{EL} of the CP-OLED-3 device. **d** Transient EL intensities of the CP-OLED-3 device when *U* was swept between zero and linearly increasing values. The exposure time to collect each data point was set at 0.01 s. **e**,**f** CPEL (**e**) and g_{EL} (**f**) spectra of the CP-OLED-3 device when the applied voltage *U* was set at the values shown in (**d**).

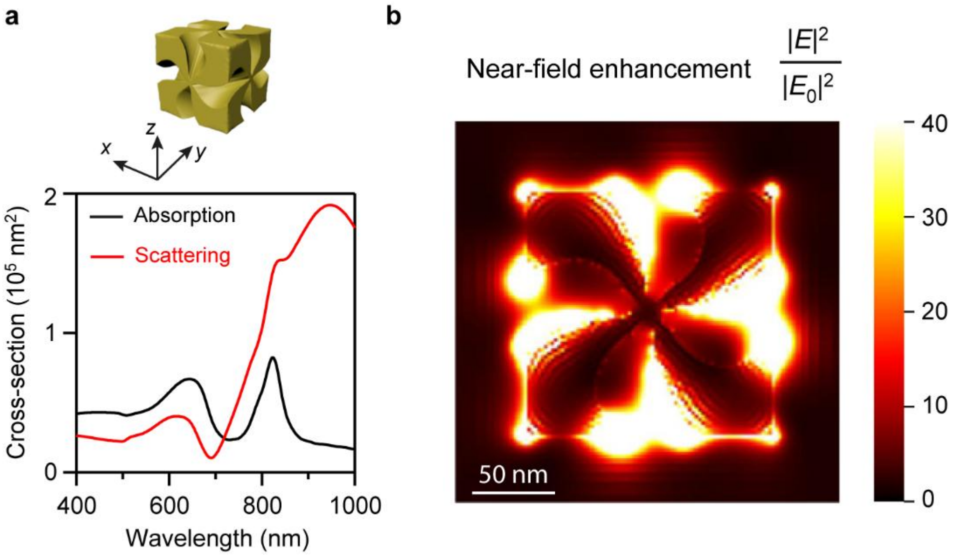

Supplementary Fig. 34 | FDTD-calculated plasmonic near-field enhancement $|E|^2/|E_0|^2$ of a chiral Au 432 helicoid III NP. a Schematic showing the 432 helicoid III NP (top) and simulation-calculated absorption and scattering spectra of the 432 helicoid III NP under the excitation of circularly polarized light. A plasmonic peak at ≈600 nm was observed from the calculated spectra, right at the CPEL emission peak of the CP-OLEDs. **b** Calculated distribution of plasmonic electric field enhancement $|E|^2/|E_0|^2$ at the surface of the Au 432 helicoid III NP when the excitation wavelength was 600 nm. The maximum plasmonic enhancement was found to be 40, located at the tips of the 432 helicoid III NP. The calculated results suggested that the plasmonic near-field enhancement from 432 helicoid III might be another reason for high EQEs of the CP-OLED-2 devices. The large plasmonic near-field of the 432 helicoid III NP can lead to a significant plasmonic Purcell effect. Excitons near the NP surface therefore decay radiatively in a faster manner. The accelerated radiative process can result in an enlarged emission quantum efficiency, and therefore give rise to higher EQEs of the chiral plasmonic NP-based CP-OLED devices.

Supplementary references

1. Yin, X. J. *et al.* Integration of fine-tuned chiral donor with hybrid long/short-range charge-transfer for high-performance circularly polarized electroluminescence. *Mater. Horiz.* **11,** 1752 (2024).
2. Meng, G. Y. *et al.* B-N covalent bond embedded double hetero-[n]helicenes for pure red narrowband circularly polarized electroluminescence with high efficiency and stability. *Adv. Mater.* **36,** 2307420 (2024).
3. Guo, W.-C., Zhao, W.-L., Tan, K.-K., Li, M. & Chen, C.-F., B, N-embedded hetero[9]helicene toward highly efficient circularly polarized electroluminescence. *Angew. Chem. Int. Ed.* **63,** e202401835 (2024).
4. Chen, Z. X. *et al*. High-performance circularly polarized electroluminescence with simultaneous narrowband emission, high efficiency, and large dissymmetry factor. *Adv. Mater.* **34,** 2109147 (2022).
5. Liao, X. J. *et al.* Planar chiral multiple resonance thermally activated delayed fluorescence materials for efficient circularly polarized electroluminescence. *Angew. Chem. Int. Ed.* **62,** e202217045 (2023).
6. Chen, Z. X. *et al.* Cascade chirality transfer through diastereomeric interaction enables efficient circularly polarized electroluminescence. *Adv. Funct. Mater.* **33,** 2215179 (2023).
7. Zhang, X. Y. *et al.* Highly efficient circularly polarized phosphorescent electroluminescence from iridium (iii) complexes with chiral ligands. *J. Mater. Chem. C* **12,** 3997–4004 (2024).
8. Wu, Z. G. *et al*. Chiral octahydro-binaphthol compound-based thermally activated delayed fluorescence materials for circularly polarized electroluminescence with superior EQE of 32.6% and extremely low efficiency roll-off. *Adv. Mater.* **31,** 1900524 (2019).
9. Yang, W. *et al*. Simple double hetero[5]helicenes realize highly efficient and narrowband circularly polarized organic light-emitting diodes. *CCS Chem.* **4,** 3463–3471 (2022).
10. Zhong, X. S. *et al*. Circularly polarized organic light-emitting diodes based on chiral hole transport enantiomers. *Adv. Mater.* **36,** 2311857 (2024).
11. Wang, Q. Y. *et al*. Constructing highly efficient circularly polarized multiple-resonance thermally activated delayed fluorescence materials with intrinsically helical chirality. *Adv. Mater.* **35,** 2305125 (2023).
12. Lu, G. Z. *et al.* Semitransparent circularly polarized phosphorescent organic light-emitting diodes with external quantum efficiency over 30% and dissymmetry factor close to 10^{-2}. *Adv. Funct. Mater.* **31,** 2102898 (2021).
13. Wan, S. P. *et al.* Axially chiral thermally activated delayed fluorescence emitters enabled by molecular engineering towards high-performance circularly polarized OLEDs. *Chem. Eng. J.* **468,** 143508 (2023).
14. Xu, Y. C., Wang, Q. Y., Cai, X. L., Li, C. L. & Wang, Y. Highly efficient electroluminescence from narrowband green circularly polarized multiple resonance thermally activated delayed fluorescence enantiomers. *Adv. Mater.* **33,** 2100652 (2021).
15. Xu, L. T. *et al*. Efficient circularly polarized electroluminescence from achiral luminescent materials. *Angew. Chem. Int. Ed.* **62,** e202300492 (2023).
16. Xie, F.-M. *et al*. Efficient circularly polarized electroluminescence from chiral thermally activated delayed fluorescence emitters featuring symmetrical and rigid coplanar acceptors. *Adv. Opt. Mater.* **9,** 2100017 (2021).
17. Tong, J. J. *et al*. Chiral sulfonyl binaphthalene-based thermally activated delayed fluorescence materials for circularly polarized electroluminescence. *Adv. Opt. Mater.* **12,** 2302730 (2024).

18. Ye, Z. Y. *et al*. Deep-blue narrowband hetero[6]helicenes showing circularly polarized thermally activated delayed fluorescence toward high-performance OLEDs. *Adv. Mater.* **36,** 2308314 (2024).
19. Yan, Z. P. *et al*. A chiral dual-core organoboron structure realizes dual-channel enhanced ultrapure blue emission and highly efficient circularly polarized electroluminescence. *Adv. Mater.* **34,** 2204253 (2022).
20. Zhang, Y.-P. *et al*. Efficient circularly polarized photoluminescence and electroluminescence of chiral spiro-skeleton based thermally activated delayed fluorescence molecules. *Sci. China Chem.* **65,** 1347–1355 (2022).
21. Yang, S.-Y. *et al*. Circularly polarized thermally activated delayed fluorescence emitters in through-space charge transfer on asymmetric spiro skeletons. *J. Am. Chem. Soc.* **142,** 17756–17765 (2020).
22. Wang, Y. F. *et al*. Chiral TADF-active polymers for high-efficiency circularly polarized organic light-emitting diodes. *Angew. Chem. Int. Ed.* **60,** 23619–23624 (2021).
23. Zhou, Y.-H. *et al*. Circularly polarised photoluminescence and electroluminescence of chiral copper(I) dimers based on *R*/*S*-2,2′-bis(diphenylphosphino)-1,1′-binaphthalene ligands. *J. Mater. Chem. C* **11,** 1329–1335 (2023).
24. Li, M., Wang, M. Y., Wang, Y. F., Feng, L. & Chen, C. F. High-efficiency circularly polarized electroluminescence from TADF-sensitized fluorescent enantiomers. *Angew. Chem. Int. Ed.* **60,** 20728–20733 (2021).
25. Tan, K.-K. *et al*. Axially chiral TADF-active materials with π-extended acceptors for highly efficient circularly polarized electroluminescence. *Chem. Eng. J.* **462,** 142123 (2023).
26. Wang, Y.-F., Li, M., Teng, J.-M., Zhou, H.-Y. & Chen, C.-F. High-performance solution-processed nondoped circularly polarized OLEDs with chiral triptycene scaffold-based TADF emitters realizing over 20% external quantum efficiency. *Adv. Funct. Mater.* **31,** 2106418 (2021).
27. Wu, X. G. *et al*. Fabrication of circularly polarized MR-TADF emitters with asymmetrical peripheral-lock enhancing helical B/N-doped nanographenes. *Adv. Mater.* **34,** 2105080 (2022).
28. Zhang, Y. P. *et al*. Circularly polarized white organic light-emitting diodes based on spiro-type thermally activated delayed fluorescence materials. *Angew. Chem. Int. Ed.* **61,** e202200290 (2022).
29. Ni, F. *et al*. Integrating molecular rigidity and chirality into thermally activated delayed fluorescence emitters for highly efficient sky-blue and orange circularly polarized electroluminescence. *Mater. Horiz.* **8,** 547–555 (2021).
30. Qu, C. *et al*. Helically chiral donor–acceptor double hetero[4]helicenes with circularly polarized thermally activated delayed fluorescence. *Adv. Opt. Mater.* **11,** 2203030 (2023).
31. Zhang, Y. P. *et al*. Chiral spiro-axis induced blue thermally activated delayed fluorescence material for efficient circularly polarized OLEDs with low efficiency roll-off. *Angew. Chem. Int. Ed.* **60,** 8435–8440 (2021).
32. Li, M. *et al*. Stable enantiomers displaying thermally activated delayed fluorescence: efficient OLEDs with circularly polarized electroluminescence. *Angew. Chem. Int. Ed.* **57,** 2889–2893 (2018).
33. Sun, B. J., Ding, L., Wang, X. Z., Tu, Z.-L. & Fan, J. Circularly polarized thermally activated delayed fluorescence OLEDs with nearly BT.2020 red emission. *Chem. Eng. J.* **476,** 146511 (2023).
34. Wang, Y.-F., Liu, X., Zhu, Y. C., Li, M. & Chen, C.-F. Aromatic-imide-based TADF enantiomers for efficient circularly polarized electroluminescence. *J. Mater. Chem. C* **10,** 4805–4812 (2022).

35. Zhang, D.-W. *et al*. D–π*–A type planar chiral TADF materials for efficient circularly polarized electroluminescence. *Mater. Horiz.* **8,** 3417–3423 (2021).
36. Tu, Z.-L. *et al*. Blue axially chiral biphenyl based thermally activated delayed fluorescence materials for efficient circularly polarized OLEDs. *Adv. Opt. Mater.* **9,** 2100596 (2021).
37. Tu, Z. L. *et al*. Axially chiral biphenyl compound-based thermally activated delayed fluorescent materials for high-performance circularly polarized organic light-emitting diodes. *Adv. Sci.* **7,** 2000804 (2020).
38. Zhao, W.-L. *et al*. Chiral thermally activated delayed fluorescence-active macrocycles displaying efficient circularly polarized electroluminescence. *CCS Chem.* **4,** 3540–3548 (2022).
39. Dong, Q. W. *et al*. Binaphthol-based chiral host molecules for efficient solution-processed circularly polarized OLEDs. *Chem. Commun.* **59,** 1473–1476 (2023).
40. Liu, T.-T. *et al*. Chiral thermally activated delayed fluorescence emitters-based efficient circularly polarized organic light-emitting diodes featuring low efficiency roll-off. *ACS Appl. Mater. Interfaces* **13,** 56413–56419 (2021).
41. Pan, Z.-P. *et al*. Configurationally stable platinahelicene enantiomers for efficient circularly polarized phosphorescent organic light-emitting diodes. *Chem. Eur. J.* **25,** 5672–5676 (2019).
42. Teng, J. M., Zhang, D. W., Wang, Y. F. & Chen, C. F. Chiral conjugated thermally activated delayed fluorescent polymers for highly efficient circularly polarized polymer light-emitting diodes. *ACS Appl. Mater. Interfaces* **14,** 1578–1586 (2022).
43. Lu, J.-J. *et al*. Pyridinylphosphorothioate-based blue Iridium(III) complex with double chiral centers for circularly polarized electroluminescence. *J. Mater. Chem. C* **9,** 5244–5249 (2021).
44. Yang, S.-Y. *et al*. A narrowband blue circularly polarized thermally activated delayed fluorescence emitter with a hetero-helicene structure. *Chem. Commun.* **57,** 11041–11044 (2021).
45. Ying, A. *et al*. Copper(I) complexes with planar chirality realize efficient circularly polarized electroluminescence. *Sci. China Chem.* **66,** 2274–2282 (2023).
46. Gu, Q. *et al*. Chiral exciplex acceptor enables circularly polarized electroluminescence with high dissymmetry factor close to 10^{-2}. *Adv. Opt. Mater.* **10,** 2201793 (2022).
47. Li, M., Wang, Y. F., Zhang, D., Duan, L. & Chen, C.-F. Axially Chiral TADF-Active Enantiomers Designed for Efficient Blue Circularly Polarized Electroluminescence. *Angew. Chem. Int. Ed.* **59,** 3500–3504 (2020).
48. Liang, Z. P. *et al*. Construction and properties of octahydrobinaphthol-based chiral luminescent materials with large steric hindrance. *Acta Chim. Sinica* **79,** 1401–1408 (2021).
49. Yan, Z.-P. *et al*. Chiral thermally activated delayed fluorescence materials based on *R*/*S*-N^2,$N^{2'}$-diphenyl-[1,1′-binaphthalene]-2,2′-diamine donor with narrow emission spectra for highly efficient circularly polarized electroluminescence. *Adv. Funct. Mater.* **31,** 2103875 (2021).
50. Wu, Z.-G. *et al*. Non-doped and doped circularly polarized organic light-emitting diodes with high performances based on chiral octahydro-binaphthyl delayed fluorescent luminophores. *J. Mater. Chem. C* **7,** 7045–7052 (2019).
51. Teng, J. M. & Chen, C. F. D-A* co-polymerization realizing chiral thermally activated delayed fluorescent polymers for highly efficient circularly polarized polymer light-emitting diodes. *ChemPhotoChem* **8,** e202300253 (2024).

52. Gong, M. H. *et al*. Planar chiral thermally activated delayed fluorescence materials based on di[2.2]paracyclophane for circularly polarized electroluminescence. *Adv. Funct. Mater.* **34,** 2314205 (2024).
53. Qian, G. W. *et al*. Chiral platinum-based metallomesogens with highly efficient circularly polarized electroluminescence in solution-processed organic light-emitting diodes. *Adv. Opt. Mater.* **8,** 2000775 (2020).
54. Yang, S. Y. *et al*. Efficient circularly polarized thermally activated delayed fluorescence hetero-[4]helicene with carbonyl-/sulfone-bridged triarylamine structures. *J. Mater. Chem. C* **10,** 4393–4401 (2022).
55. Qu, L. *et al*. Axially chiral biphenoxazine-based thermally activated delayed fluorescence materials for solution-processed circularly polarized organic light-emitting diodes. *Chem. Eng. J.* **471,** 144709 (2023).
56. Sun, S. B. *et al*. Thermally activated delayed fluorescence enantiomers for solution-processed circularly polarized electroluminescence. *J. Mater. Chem. C* **7,** 14511–14516 (2019).
57. Tian, Y. *et al*. Host molecule enhanced aggregation induced emission of chiral silver nanoclusters for achieving highly efficient circularly polarized electroluminescence. *Nano Res.* **16,** 7733–7741 (2023).
58. Song, F. Y. *et al*. Highly efficient circularly polarized electroluminescence from aggregation-induced emission luminogens with amplified chirality and delayed fluorescence. *Adv. Funct. Mater.* **28,** 1800051 (2018).
59. Chen, L., Zhang, Y., Ye, S.-H. & Zheng, W.-H. Planar chiral [2.2] paracyclophane-based thermally activated delayed fluorescent materials for circularly polarized electroluminescence. *ACS Appl. Mater. Interfaces* **13,** 25186–25192 (2021).
60. Jiang, A. W. *et al*. Functionalization of the octahydro-binaphthol skeleton: A universal strategy for directly constructing D–A type axially chiral biphenyl luminescent molecules. *J. Org. Chem.* **89,** 3605–3611 (2024).
61. Teng, J.-M. & Chen, C.-F. Chiral TADF polymers realizing highly-efficient deep-red circularly polarized electroluminescence over 660 nm. *Adv. Opt. Mater.* **11,** 2300550 (2023).
62. Luo, X.-F. *et al*. Multicolor circularly polarized photoluminescence and electroluminescence with 1,2-diaminecyclohexane enantiomers. *ACS Appl. Mater. Interfaces* **12,** 23172–23180 (2020).
63. Zhang, Y., Li, D., Li, Q. H., Quan, Y. W & Cheng, Y. X. High comprehensive circularly polarized electroluminescence performance improved by chiral coassembled host materials. *Adv. Funct. Mater.* **33,** 2309133 (2023).
64. Li, Z.-Q. *et al*. Electrically amplified circularly polarized luminescence by a chiral anion strategy. *Angew. Chem. Int. Ed.* **62,** e202302160 (2023).
65. Zhang, X. Y. *et al*. High brightness circularly polarized blue emission from non-doped OLEDs based on chiral binaphthyl-pyrene emitters. *Chem. Commun.* **55,** 9845–9848 (2019).
66. Geng, Z. X. *et al*. Inverted and amplified CP-EL behavior promoted by AIE-active chiral co-assembled helical nanofibers. *Adv. Mater.* **35,** 2209495 (2023).
67. Jiang, Z. Y. *et al*. Rational design of axially chiral platinabinaphthalenes with aggregation-induced emission for red circularly polarized phosphorescent organic light-emitting diodes. *ACS Appl. Mater. Interfaces* **12,** 9520–9527 (2020).
68. Liu J. X. *et al*. A circularly polarized (CP) white organic light-emitting diode (WOLED) based on a chiral organo-Sm^{3+} complex. *J. Mater. Chem. C* **11,** 1265–1270 (2023).

69. Wang, L. *et al*. Axially chiral bis-cycloplatinated binaphthalenes and octahydro-binaphthalenes for efficient circularly polarized phosphorescence in solution-processed organic light-emitting diodes. *Inorg. Chem.* **60,** 13557–13566 (2021).
70. Zhang, X. Y., Xu, Z. R., Zhang, Y., Quan, Y. W. & Cheng, Y. X. High brightness circularly polarized electroluminescence from conjugated polymer F8BT induced by chiral binaphthyl-pyrene. *J. Mater. Chem. C.* **8,** 15669–15676 (2020).
71. Wang, M. Y. *et al*. Chiral helical polymer-induced efficient circularly polarized organic light-emitting diodes. *Adv. Opt. Mater.* **12,** 2301513 (2023).
72. Zhang, Y., Li, J., Quan, Y. W., Ye, S. H. & Cheng, Y. X. Solution-processed white circularly polarized organic light-emitting diodes based on chiral binaphthyl emitters. *Chem. Eur. J.* **27,** 589–593 (2021).
73. Frédéric, L. *et al*. Maximizing chiral perturbation on thermally activated delayed fluorescence emitters and elaboration of the first top-emission circularly polarized OLED. *Adv. Funct. Mater.* **30,** 2004838 (2020).
74. Zhang, Y., Li, Y. P., Quan, Y. W., Ye, S. H. & Cheng, Y. X. Remarkable White Circularly polarized electroluminescence based on chiral co-assembled helix nanofiber emitters. *Angew. Chem. Int. Ed.* **62,** e202214424 (2023).
75. Zhang, X. Y., Xu, Z. R., Zhang, Y., Quan, Y. W. & Cheng, Y. X. Controllable circularly polarized electroluminescence performance improved by the dihedral angle of chiral-bridged binaphthyl-type dopant inducers. *ACS Appl. Mater. Interfaces* **13,** 55420–55427 (2021).
76. Geng, Z. X., Zhang, Y. X., Zhang, Y., Quan, Y. W. & Cheng, Y. X. Amplified circularly polarized electroluminescence behavior triggered by helical nanofibers from chiral co-assembly polymers. *Angew. Chem. Int. Ed.* **61,** e202202718 (2022).
77. Dhbaibi, K. *et al*. Achieving high circularly polarized luminescence with push–pull helicenic systems: from rationalized design to top-emission CP-OLED applications. *Chem. Sci.* **12,** 5522–5533 (2021).
78. Zinna, F. *et al*. Design of lanthanide-based OLEDs with remarkable circularly polarized electroluminescence. *Adv. Funct. Mater.* **27,** 1603719 (2017).
79. Zinna, F. *et al*. Modular chiral Eu(III) complexes for efficient circularly polarized OLEDs. *J. Mater. Chem. C* **10,** 463–468 (2022).
80. Zinna, F., Giovanella, U. & Di Bari, L. Highly circularly polarized electroluminescence from a chiral europium complex. *Adv. Mater.* **27,** 1791–1795 (2015).
81. Chen, J. *et al.* An unprecedented spike of the electroluminescence turn-on transience from guest-doped OLEDs with strong electron-donating abilities of host carbazole groups. *Mater. Horiz.* **8,** 2785–2796 (2021).
82. Wan, L., Liu, Y. Z., Fuchter, M. J. & Yan, B. H. Anomalous circularly polarized light emission in organic light-emitting diodes caused by orbital–momentum locking. *Nat. Photonics* **17,** 193–199 (2023).
